# Redox-Regulated Nonvolatility Switching in Single-Nanopipette Memory Diodes

**DOI:** 10.1021/jacs.6c10813

**Published:** 2026-09-19

**Authors:** Dipak Baram, Ruoyu Yang, Gangli Wang

**Affiliations:** Department of Chemistry, 1373 Georgia State University, Atlanta, Georgia 30302, United States

## Abstract

Nanofluidic memory devices featuring brain-mimicking functions could mitigate von Neumann architecture limitations, but long-term memory and controllable volatility–nonvolatility state switching remain challenging to establish, which are foundational for higher-order complexity functions. By integrating the redox polymer PEDOT:PSS into single-nanopipette diodes with memristor properties, hysteretic ion transport is regulated by coupled electron transfers, which switch nanotip conductivity hundredfold. Oxidation and reduction of PEDOT lock in different nanotip conductivity states, accompanied by stochastic current spikes and switching behavior. Reversible volatile and nonvolatile rectified memory effects are controlled by low electric potentials: redox-regulated conductivity state changes drive long-term memory and potential sweeping or pulse trains stimulate short-term ionic memory effects. Integrating gate controls in three-terminal transistors into two-terminal memristor diodes constitutes a generalizable design principle for advancing iontronics and neuromorphic computing capabilities.

## Graphical abstract

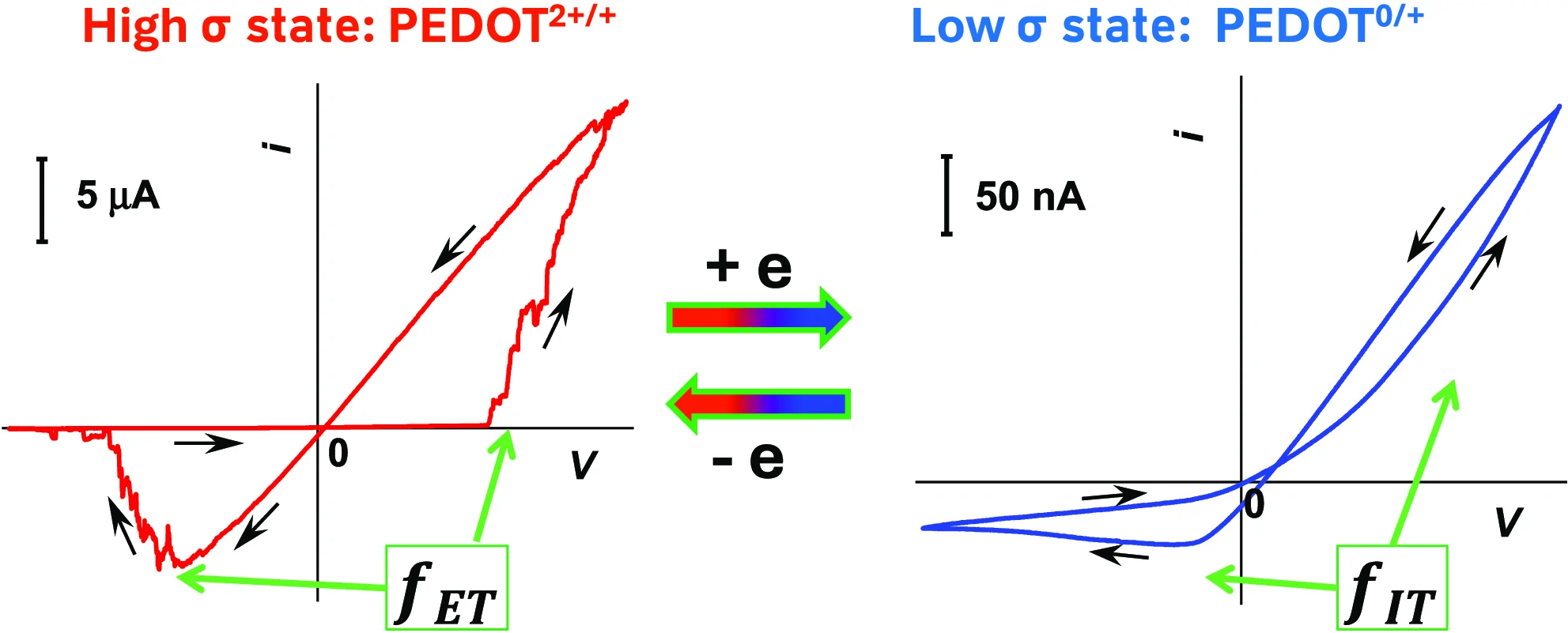

## Introduction

Tremendous energy efficiency in biological synapses and neurons is achieved through an organically integrated network in the biological matrix for information processing and transmission.^1,2^ A bottleneck in artificial systems, estimated to be 10 ^8,9^ less efficient, is the memory wall, i.e., data exchange between solid-state memories and processors in the von Neumann architecture.^3^ Memristors and broadly defined electronic devices with memory effects offer intrinsic advantages to mitigate such limitations in that their conductance “memorizes” the previous states, enabling integrated logic operation and memory functions.^4–9^ Distinct from the three classic passive circuit elements, memristors constitute a fourth type of two-terminal electronic devices, which have been categorized or further extended into memtransistors, memcapacitors, and meminductors based on the phase shift relative to a stimulating electric field.^10,11^ After being conceptualized by Chua in 1971 and realized by Williams and co-workers in 2008, a variety of mechanisms have been devised to gain memristor properties,^6,12^ overwhelmingly focused on solid-state materials and devices. Technical barriers such as scalability, device-to-device variation, drift and nonlinearity issues, and high energy costs root from fundamental thermodynamic limitations, such as entropy factors in the localized filament (de)­formation, phase transitions, valence state changes, and chemical transformations, under the stimulation of temperature variations, electrochemical reactions, and additionally electrical, magnetic, or mechanical fields.^13–17^

Nanofluidics with aqueous electrolytes better mimic biological systems and emerge as a new route toward developing advanced memristor functions for iontronics, neuromorphic computing, ion enrichment and energy applications, and emulating neuron and brain functions.^18–26^ Achieving nonvolatility or long-term memory and functions of high complexity along with scale-down to submicrometers remains challenging in fluidic and electrochemical systems.^27–29^ Fundamentally, the memory effect in nanofluidic devices originates from and is characterized by the hysteresis in the often rectified ionic current (ICR).^25,30–32^ The mechanism and operational principles based on nanoscale interfacial processes, versus bulk and in solid states, offer intrinsic advantages in greater reversibility and consistency of the properties, scalability from bottom-up synthesis, versatility to address device material variability, and low power consumption. Our reports on pinched current–potential (*I*–*V*) loops with a non-zero crosspoint, a signature of memristors, in ion transport (IT) through single conical nanopores,^33–35^ along with the inductive behaviors in electrochemical impedance spectroscopy analysis,^34,36^ corroborate well with independent molecular dynamics simulation revealing memcapacitive features.^37^ Dynamic transitions from higher to lower conductivity states in ICR are associated with the release of enriched charge carriers and can display strong negative differential resistance (NDR) in the dynamic nanofluidic system, which offer promising avenues toward applications such as preconcentration.^38–40^ Alternatively, steady-state or stationary NDR has been theorized to enable Hopf bifurcation in a single nanopore as a minimum artificial neuron displaying spiking electrical signals consistent with the Hodgkin–Huxley neural model.^41–43^ In addition to simple aqueous electrolytes, peptides, lipids, and other chemical modalities have been incorporated into nanofluidic memristor systems, tailored toward different performance matrices for neuromorphic and other applications.^44,45^ To gain controllable state switching and higher-order complexity functions without introducing excessive state variables or complicated material device designs, herein, we functionalize single conical quartz nanopipettes with the redox polymer PEDOT:PSS (poly­(2,3-dihydrothieno-1,4-dioxin)-poly­(styrenesulfonate)). Extraordinarily reversible and consistent long- and short-term memory as well as bursting and switching-type advanced functions are achieved through coupled electron transfer (ET) and hysteretic IT. By tuning the potential waveform (*V*_*x*_,*t*), the doping level of PEDOT:PSS is adjusted by ET reactions (set of state variables *f*_ET_), which in turn modulates the conductivity and IT dynamics (set of state variables *f*_IT_). With electrodes in direct electric contact with PEDOT:PSS, the gate function used in three-terminal field-effect transistors is incorporated into a two-terminal ionic nanofluidic memristor diode where the electrodes serve dual purposes in driving ET and IT. Successful regulation of volatile and nonvolatile memory behaviors via redox chemistry establishes a greatly simplified and generalizable design principle.

## Results and Discussion

### ET and IT in PEDOT:PSS-Derivatized Single Conical Nanopipettes (NPs)

Individual nanopipettes loaded with PEDOT:PSS aqueous solution, referred to as PEDOT@NP, are crosslinked under heating overnight.^46,47^ Fig. 1 shows sketches representative of electrode configurations for through-nanopore ion transport (IT) measurements, where the same PEDOT:PSS is crosslinked outside simultaneously. Optical images of customized gadgets and the device setup are provided in Fig. S1. Scanning electron microscopy (SEM) images in Fig. 1B show an intact nanotip (i) and a broken one (iv), revealing the continuous polymer filling at the nanotip and interior. Energy dispersive X-ray (EDX) spectroscopy captures sulfur and carbon signals, further confirming the presence of PEDOT:PSS, in reference to the Si and Al signals from the quartz capillary and aluminum holder, (ii) and (v). The side view of a PEDOT@NP (iii) illustrates the conical geometry along with an empty nanotip (vi), where no S signal was detected in EDX. The consistency of the PEDOT@NP synthesis is supported by additional examples and analysis in Fig. S2.

**Figure 1:**
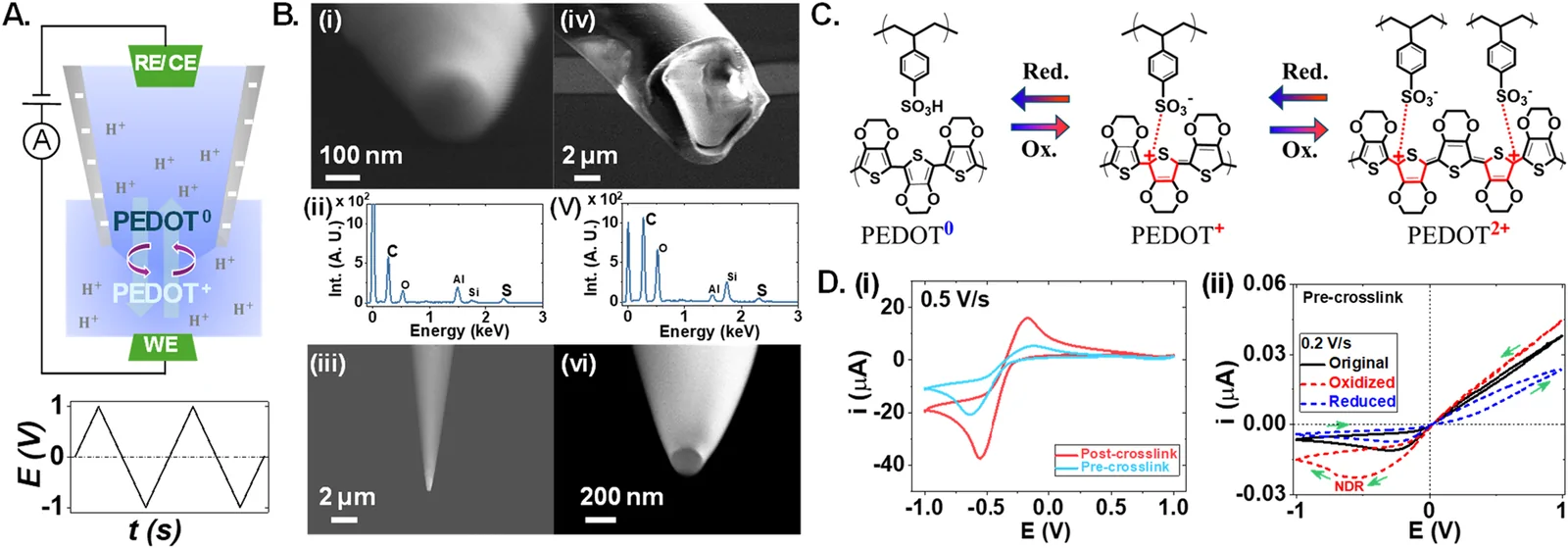
Overview of the experimental system. (A) Electrode layout and triangular potential waveform for CV and ion transport measurements. An Ag/AgCl (or Pt) wire as a working electrode (WE) is outside of a nanopipette against another Ag/AgCl (or Pt) wire as reference/counter electrodes, with the RE/CE inside the capillary. Note that the RE/CE is always inside the NP and shorted due to the limited space unless otherwise specified (WE and RE/CE as a two-electrode setup, 2-E). The electrodes are in direct electrical contacts with PEDOT to drive faradaic reactions. (B) SEM and EDX analysis of PEDOT-loaded nanopipettes. When preparing the nanotips for SEM/EDX, no PEDOT:PSS solution was added outside of the nanopipette during cross-linking. (C) Molecular structures and sequential electron transfer (ET) reactions. Ox. and Red. refer to oxidation and reduction, respectively. (D) (i) Cyclic voltammograms (CV) of PEDOT:PSS in the bulk from a Pt WE (no NP). (ii) Characteristic ionic memristor-type current–potential (*I*–*V*) features from pre-crosslinked PEDOT@NP. Data collected with the 2-E setup from the original and further oxidized and reduced PEDOT via 90-second electrolysis under +/– 0.9 V (details in the SI).

ET and IT features of PEDOT, in the bulk and with NP confinement, are compared in Fig. 1 C and D. Using a Pt WE for the bulk sample, a prominent cathodic peak in CV at about −0.55 V corresponds to the reduction into PEDOT^0^ (Di). Two anodic processes are observed, the first being the reversal to PEDOT^+^ and another much weaker and irreversible one at around +0.65 V related to the further oxidized PEDOT^2+^.^48^ These features are better resolved by differential pulse voltammograms and CVs at additional scan rates in Fig. S3. The open-circuit potential is about +0.4 V prior to cross-linking and about +0.3 V after. The results further reveal PEDOT^+^ as the dominant species mixed with a lower amount of bipolaron PEDOT^2+^. Despite the chemical composition being unavailable for the commercial material (Heraeus), PSS is known to have much higher molecular weight over PEDOT especially prior to cross-linking. With the solution pH at about 2 prior to cross-linking, the proton is considered the counterion associated with electron transfer (ET). Note that the counterion identity may influence the quantitative transport characteristics, but the underlying mechanism established herein is expected to remain valid regardless of the specific counterion type. The faradaic current increases upon cross-linking, consistent with the reported increase in the conductivity of crosslinked Clevios PEDOT:PSS at hundreds to thousands of S/cm, generally attributed to the hole doping and formation of PEDOT nanodomains in the negatively charged PSS framework.^46,47^

Faradaic features are unresolved when pre-crosslinked PEDOT solution is tested in single NPs. This is because the applied potential primarily drops at the nanotip region due to the conical geometry; consequently, ET can no longer be driven effectively at the WE interface. The recorded *I*–*V* curves are limited by IT across the nanotip, displaying intense ICR, hysteretic pinched *I*–*V* loops with a non-zero crosspoint, and NDR (Dii). Steady-state ICR has been observed in various nanopores and nanofluidic channels, mostly with aqueous electrolytes.^49–51^ The hysteresis and NDR features reveal the dynamics of the ICR. All three phenomena arise from the dynamic IT at the nanoscale interfaces limited by the smallest conical nanotip region.^30,42,50,51^ However, the widely accepted mechanism in electrostatic effects from device surface charges is not applicable because the high density of space charges of PEDOT:PSS would suppress the electrostatics and result in linear ohmic *I*–*V* behaviors, similar to high ionic strength effects of, e.g., 1 M KCl. A mechanism section at the end further explains coupled ET and IT. The IT-dominated features here can be explained by the gradient(s) distribution of space charge at the most restrictive conical nanotip region, formed according to the strong electric field established by the potential bias. Accordingly, the non-zero crosspoint is an intrinsic property of the system reflecting the effective trans-nanopore potential arising from the intrinsic electric field.^30,42,50,51^ From the molecular perspective, the relatively immobile PSS defines a negative framework, within which proton and possible sodium ions from the commercial PEDOT:PSS (and/or the formation of PEDOT^2+/+^ via ET reactions elaborated later) serve as mobile charge carriers. The space charge mechanism finds support from similar IT features resulting from inhomogeneous distribution of space charges in the barrier oxide layer of the anodized aluminum oxide membrane^53,54^ and polymer-derivatized micropipettes and pores.^23,55^ The transport symmetry is further broken by the radial influx versus planar efflux,^33,38,39^ a signature of nanoelectrodes over macroscopic ones. To echo the electrolysis of crosslinked PEDOT@NP discussed next, ITs of pre-crosslinked PEDOT aqueous solution in the same nanopipette, further oxidized and reduced separately, are compared in (Dii). The current increases slightly with oxidation and decreases by reduction electrolysis, but all three *I–V* features, ICR, hysteretic loops, and NDR, are qualitatively retained.

Comprehensive control experiments summarized in Fig. S4 and explanations eliminate the uncertainties from electrode configurations: (1) switching the WE from the outside to the inside of the capillary flips the polarities of rectification and hysteresis loops; (2) Pt or Ag wires as polarizable and nonpolarizable electrode materials induce insignificant changes in those current features; and (3) the key trends remain unchanged with separated electrodes for current and voltage controls with the 3-E or 4-E setup. Collectively, the results strongly suggest the insignificant roles of ET kinetics or the EDL effects at the macroscopic electrode:polymer interfaces away from the nanopore. In other words, these memristor current features are limited by asymmetric nanotip confinement. Altogether, when confined inside NPs, especially after PEDOT cross-linking, the through-nanopore current is restricted by the smallest nanotip: IT through the polymer framework and ET for (de)­doping of PEDOT at suitable potential.

### Short-Term Memory Effects Stimulated by Pulsed and Continuous (Within Each Pulse) Potential Waveforms

With the hysteretic IT properties observed (e.g., Fig. 1 Dii), the short-term plasticity of crosslinked PEDOT@NP is demonstrated by potentiation and depression, i.e., increase and decrease in current under repeated potential pulses, respectively. Representative results from two pulse potentials and several pulse intervals are compared in Fig. 2. Starting from zero to more positive potential, higher conductivity is built up over time, as shown in panels A and C. Under a positive voltage pulse, mobile cations are enriched in the negative polymer framework at the conical nanotip via concentration polarization, increasing the local ionic conductivity (potentiating). In earlier vs later pulses, e.g., #1 vs #50, current increases more prominently. Within each pulse, the increase is continuous under a continuous stimulus until a steady state can be reached under the constant potential. Shorter pulse intervals *t*_G_, curves in (Ai) vs (Aii), allow for faster potentiation to reach higher conductivity states. By plotting the last point in each pulse, the inverse dependence of conductance on *t*_G_ over increasing pulse number is obvious (Aiii and Ciii). At higher pulse amplitudes (A vs C), the trends are the same, but different sets of conductance states are reached.^14^

**Figure 2:**
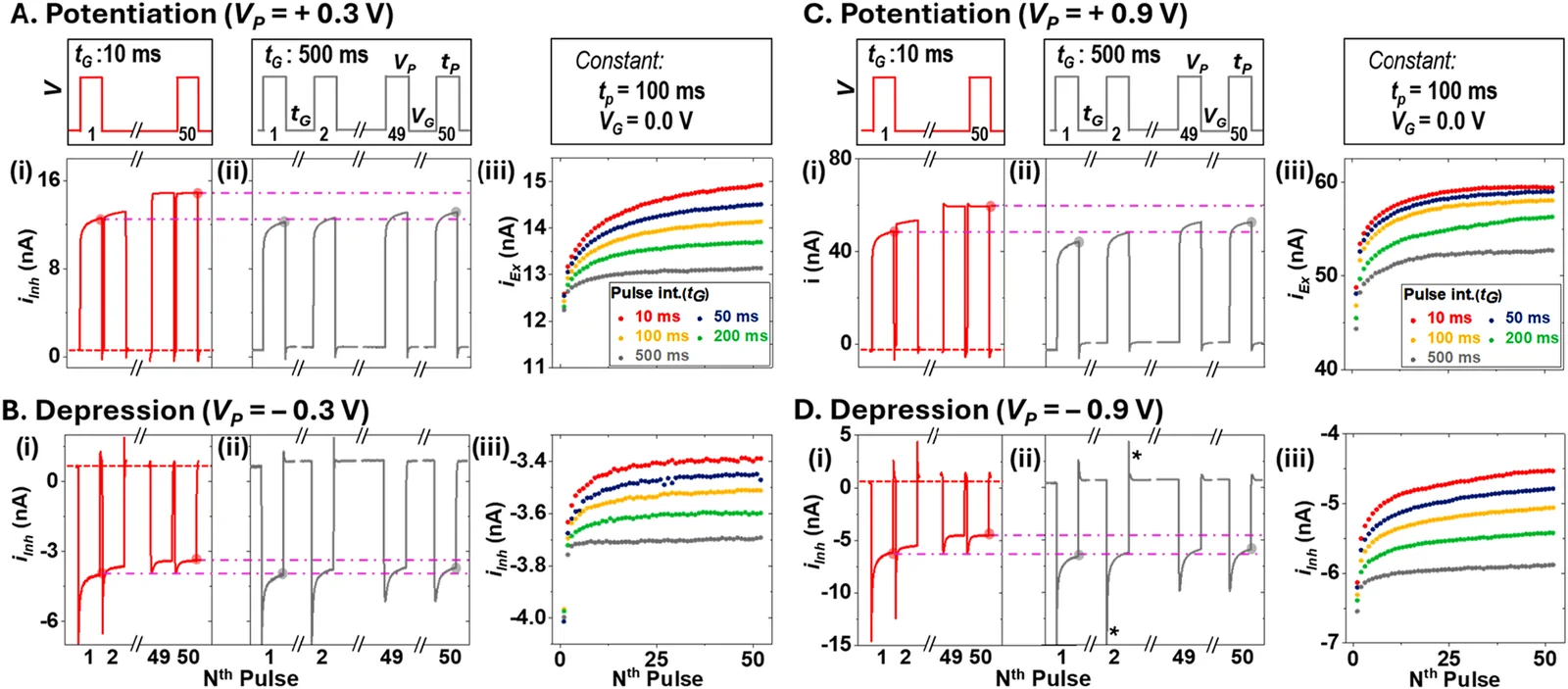
Excitatory (*top, +V*) and inhibitory (*bottom, −V*) conductance features of crosslinked PEDOT@NP under pulse train stimulus. The pulse duration is 0.1 s with +/–0.3 V pulse amplitude (*V*_*P*_) in (A, B) and +/–0.9 V in (C, D). The potential is kept constant at 0.0 V during pulse intervals/gaps (*t*_G_). Current is recorded at 1 ms per point. The first and last two pulses (out of 50) are enlarged, respectively, in (i) & (ii) with different pulse intervals (*t*_G_) to illustrate consistency and changes in individual pulses. The trend over repeated pulses is shown in (iii) by plotting the last points in each pulse against the pulse number because full data obscure the detailed features. Pulse train at each *t*_G_ was preconditioned at 0.0 V for 10 s, and data were collected in a sequence, from higher to lower *t*_G_. * indicates RC charging spikes associated with each potential step (not all charging spikes are labeled for brevity).

Pulses to negative potentials cause depression toward lower conductance states, summarized in (B, D). Other than the current decrease (amplitude approaching zero) instead of increase, the dependence on the potential waveform is the same as potentiation. Both cases demonstrate short-term plasticity or memory tunable by the stimulating potential waveform, where a later current response is built on the conductance established by the previous pulse, partially dependent on the losses of concentration polarization at the transport-limiting nanotip region during the pulse interval when a zero bias is applied. It is worth noting that those IT properties are not exclusive to the exact pulse parameters. By varying pulse parameters shown in Fig. S5, additional modulation kinetics are achieved at a faster/slower rate but display the same trends. For reference, pre-crosslinked PEDOT@NPs are confirmed to display similar short-term memory effects (Fig. S6). However, its relatively low (electrical) conductivity prohibits the ET controls introduced next and thus is not further studied.

Unlike binary digital systems, multiple discrete conductance states are easily accessible by simply tuning the applied potential waveform, reminiscent of the multiple levels of conductivity states in neurons. The pulse paired facilitation or depression (PPF or PPD) ratio is analyzed in Fig. S7 A using the first two consecutive current peaks (*I*_2_ and *I*_1_ in eq 1).^56^

$$\mathrm{PP}F\;\mathrm{or}\;\mathrm{PPD}\;\%=\left|\frac{I_{2}-I_{1}}{I_{1}}\right|\times 100$$ 1

2 $$y=A\times e^{-t_{G}/\tau }+B$$

3 $$y=A\times \left(1-e^{-t_{G}/\tau }\right)+B$$

A single exponential decay fits the PPF/PPD trend adequately. An additional exponential term will marginally improve the fitting and be consistent with the biexponential approach in literature practice, but it is not a necessity in our well-defined chemical system and thus not included.^56^ Within individual pulses, an exponential decay is used for depression at LC states, characterized by the universal RC time constant when an electrochemical interface is stimulated by a simple potential step, eq 2.^57^ RC stands for resistance capacitance, commonly referred to as time constant τ; A and B are constants accommodating experimental settings and errors. The exponential decay function is rewritten (eq 3) to fit the potentiation in HC states, in which the negative sign is based on the opposite phase shift of the inductive element with respect to the applied electric field and nanopore equivalent circuit models.^5,32,34^ In other words, eqs 2 and 3 are basically the RC exponential terms accommodating the intrinsic electric field of the nanotip instead of the applied field: exponential decay in LC (when the intrinsic and applied electric field are in the same direction, i.e., a capacitive phase shift) and exponential growth in HC (when the two field directions are opposite, i.e., an inductive phase shift).

The time constant is about 74 ms from PPF and 25 ms from pulse paired depression (PPD) fitting against pulse intervals (Fig. S7 A). Noteworthily, the initial current spikes denoted by * in Fig. 2, arising from universal RC charging from any potential steps, is excluded because it is nonspecific to or irrelevant to the nanotip PPF/PPD (although a second exponential term can be introduced for it). Fittings of the current profiles in individual pulses (Fig. S7 B) generate a time constant of 36 ms for facilitation and 14 ms for depression, quite consistent with the PPF and PPD from repeated cycles. High-quality fitting analysis using a simple single exponential function strongly supports our interpretation of IT dynamics. The analytic approach introduced here is believed to be universally applicable and superior over otherwise phenomenological treatments. Excitingly, despite operating within a polymer framework and under a different governing mechanism, these time constants correlate reasonably with the recent model established by Bisquert, Bou, and co-workers,^58^ in that the relaxation time increases with the extent of enriched charge carriers and then decreases, forming a bell shape trend (18 ms at −0.9 V, 14 ms at −0.3 V, 36 ms at +0.3 V, then 28 ms at +0.9 V; data from the first current pulse in each series). Empowered by these quantitative analysis approaches, the time constant(s) or dynamics is tunable and predictable by ionic strength and device parameters.^58^ These results demonstrate controllable analog (continuous in one pulse, short or long) and digital (repeated pulses) transitions to different conductance states.

### Stochastic Current Spikes and Switching between High- and Low-Conductivity (σ) States

The conductivity of PEDOT@NP increases by orders of magnitude with cross-linking over time (Fig. 3A), following the literature of DMSO-assisted cross-linking conditions.^46,47,59^ Besides retaining ICR, hysteretic *I–V* loops, and NDR, transient current spikes and switching to very high-current, high*-σ* states are observed in HC states toward the end of the cross-linking reaction (e.g., 16 to 18 h). As illustrated in Fig. 3B, individual spikes occur when the potential scans positive, stochastically over repeated cycles but within a narrow potential range. The current spikes shift to more positive potential and become more frequent at higher scan rates. ICR, hysteresis, and NDR diminish at higher scan rates due to less time allowed for concentration polarization–redistribution of mobile ions.^32,42^

**Figure 3:**
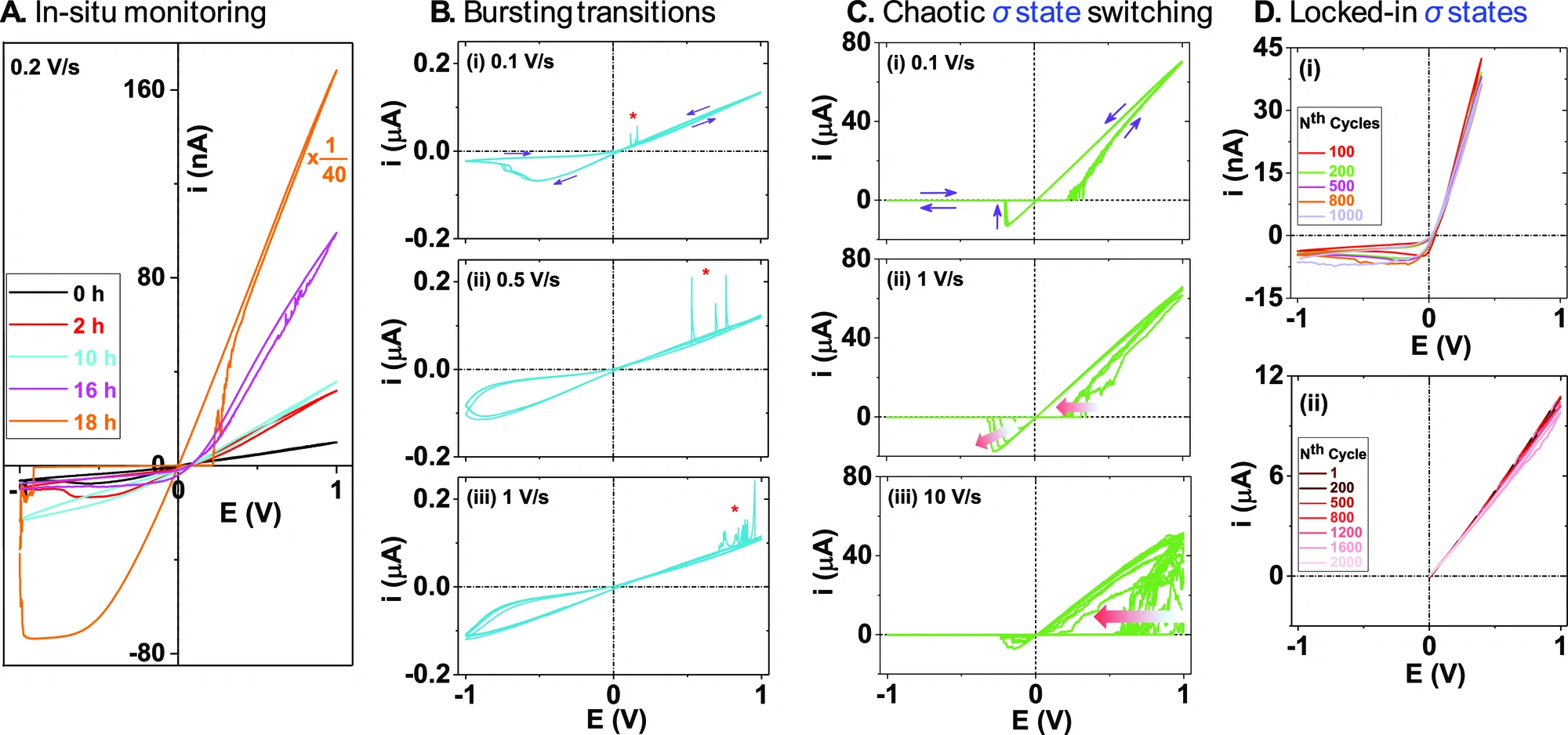
Transitions between high- and low-conductivity (*σ*) states of PEDOT@NP measured by CV at varied scan rates and potential range. (A) Room temperature *I*–*V* curves collected periodically during cross-linking. Note that current at 18 h was divided by 40 to be in the same scale. (B) Emerging transient current spikes (indicated by red asterisks) toward the end of cross-linking (16–18 h). Four *I*–*V* cycles are shown at each scan rate. Arrows in panel (i) show the directions of potential scan (the same in other panels). (C) Discrete conductivity switching after complete cross-linking. Note that current at low*-σ* states is not zero and reminiscent of those in (B). Block arrows indicate trends with increasing scan cycles (four cycles shown for 0.1 and 1 V/s; 10 for 10 V/s). (D) Locked-in low*-σ* (i) and high*-σ* (ii) states within narrower potential ranges after electrolysis at −0.9 V and +0.9 V/s, respectively. The scan rate is 0.2 V/s over thousands of cycles with insignificant current drift (5–7 cycles shown for clarity).

Persistent switching behaviors and prolonged stay in high (or low)*-σ* states (Fig. 3C) are achieved after complete cross-linking, i.e., conductivity stabilizes after 18 h. The current in the negative potential range remains mostly low with amplitude and RC loop comparable to those in panel B. When the potential scans adequately positive past the cross point *V*_CP_, the current increases sharply by two orders of magnitude. In the reverse scan to negative, the current decreases abruptly past the cross point at around −0.1 V to −0.2 V, switching back to the low*-σ* states. In general, the current branches from −1.0 V (at low*-σ* states) to HC states and from +1.0 V (at high*-σ* states) to LC states are highly stable and consistent in multiple cycles and different scan rates, until switching occurs. Phenomenologically, these features (more results in Fig. S8–S9) are reminiscent to those, e.g., filament formation in solid-state memristors. The current changes herein result from faradaic ET through which oxidation increases the PEDOT hole doping level and thus conductivity, while reduction does the opposite. Two processes account for these observations further explained in the mechanism section: gradual formation of an interconnected PEDOT nanodomain within the PSS framework and increase in doping levels.^46,47,59^

The highly reversible switching behaviors vary in the potential range and appear stochastic. At low scan rates shown in C­(i), the ET-induced switching potentials to high- and low*-σ* states, *V*_S‑>H_ and *V*_S‑>L_, have a narrower variation over multiple scans. At a high scan rate of 10 V/s, *V*_S‑>H_ shifts significantly positive in earlier scans and back in later scans, while *V*_S‑>L_ is much closer to the cross point. Features at 1 V/s are intermediate with transition from low- to high-scan rates, albeit the *V*_S‑>L_ appears to shift negatively over multiple scans. Detailed analysis of the nonlinear dynamics of the coupled ET–IT is beyond the scope of this report, but the potential profiles and charge accumulation (and depletion) are believed to be the dominant factors. For example, a higher scan rate means less time for concentration polarization (less rectification and less hysteresis) and thus less enriched ions or less current increase when the potential scans positive. Accordingly, a higher overpotential or longer time is necessary to initiate the switching to high*-σ* states. Over repeated scans, more charges are accumulated and the switching potential shifts back. The switch to low*-σ* states is less visually straightforward as it depends on the enriched charges, area between the two current branches in high*-σ* states divided by scan rate (A*V/(V/s) = C), the same principle of NDR effects as previously reported.^38,39^

Knowledge of the switching potential range allows us to selectively trigger (or exclude) *V*_S‑>H_ or *V*_S‑>L_ by applying e.g., +/- 0.9 V. PEDOT@NP is therefore locked to low*-σ* or high*-σ* states (Fig. 3D). Impressively, both high- and low*-σ* states are sustained over thousands of potential scan cycles. Although we cannot approach negative potentials in high*-σ* states (to avoid switching), *I*–*V* curves of PEDOT@NP in low*-σ* states can go up to +0.4 V in which robust ICR, hysteresis, and NDR are observed consistently. Additional scan rate results and data from additional devices in Fig. S8–S9 demonstrate high fidelity in reproducibility and controllability. The (a)­periodic bursting and adaptation-type state switching behaviors, characteristics of advanced neuronal functions, are driven by ET reactions, which regulated the coupled IT properties. The additional state variables of *f*_ET_ provide the controls on the opposing processes required for bifurcation based on classic electrochemical oscillation systems: dynamic high conductivity doping and PEDOT nanodomain formation driven by ET as accelerators versus inhibiting dissipation such as phase segregation or dedoping. The ET-induced switching differs fundamentally from the pure IT-based NDR and memristor properties in low*-σ* states or other systems.

### Long-Term Memory (LTM) Effects in PEDOT@NP

To evaluate the LTM, conductance states are monitored by consecutive read pulses over hours and compared in Fig. 4, after the PEDOT@NP is electrolyzed to high*-σ* states via Write at +0.9 V in reference to those from the low*-σ* states (Final Read versus Initial Read). The two-orders-of-magnitude higher conductance persists over hours, a drastic improvement over the state of the art by several minutes.^27–29^ To further demonstrate that the LTM is not due to the applied read potential, repetitive final read results, with hour-long intervals instead of continuous recording, are provided in Fig. S10. The current fluctuation increases slightly after 3 h, but the high*-σ* states hold. The slight current increase within each pulse (inset panels) falls into the short-term effects shown in Fig. 2 and associated discussion. The gradual current increase over time/cycle and current fluctuation during final read at high *σ* states are likely associated with the polymer-phase dynamics discussion in the mechanism section. It is worth noting that we do not believe that those are noise or uncontrolled drift, which is common for long recording, based on the consistent baseline current at both high- and low*-σ* states shown at the bottom.

**Figure 4:**
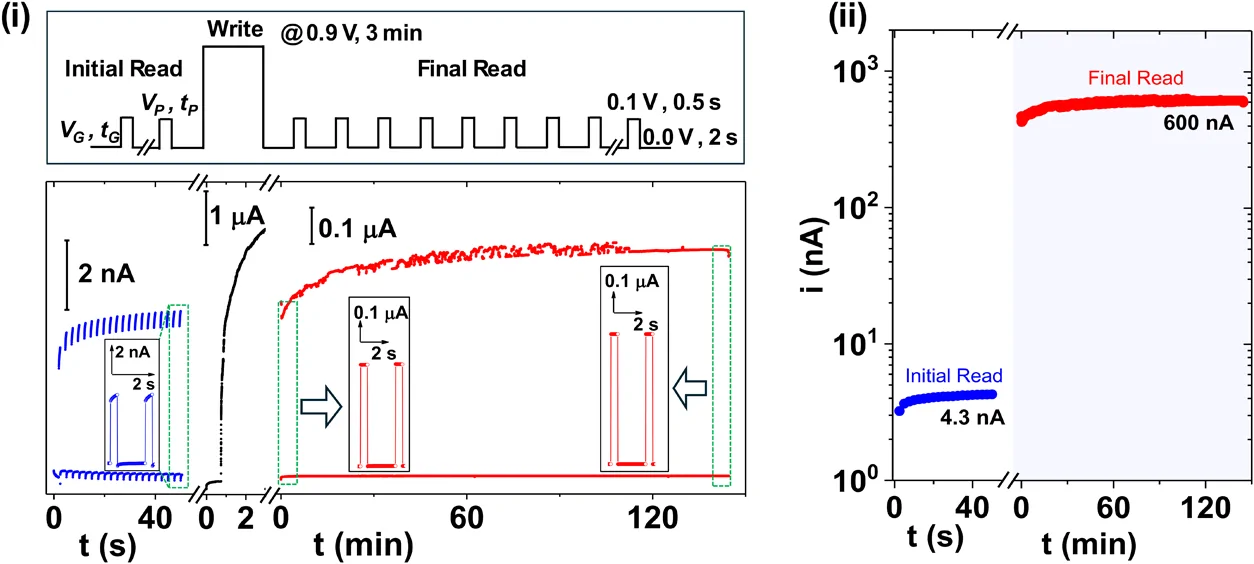
Characterization of LTM in PEDOT@NP. (i) Read–Write–Read pulse waveform (top) and the corresponding current–time trace (bottom) including 2 h consecutive Final Read pulses. Three insets are enlarged views of the last two pulses from Initial Read and the first and last two pulses from the Final Read. Note that the scales for current and time axis are different for the three steps; and Initial and Final Reads are separated by breaks from current during Write at much higher potential bias. Current sampling rate is 20 ms per point, with the pulse duration of 0.5 s at a Read potential of +0.1 V. Baseline current during a pulse interval/gap of 2 s at 0.0 V is also provided to demonstrate the consistency over the long recording. A −0.9 V electrolysis for 50 s preconditions the system to low conductivity as the initial state. (ii) Simplified view of the conductance states before and after the Write at +0.9 V. The last point during each Read pulse is plotted in log scale for direct comparison.

### Stability and Reversibility by Electrolysis as a Gating Function

The results in Fig. 5 demonstrate controllable switching between the high- and low*-σ* states, hundredfold different, in the two-terminal PEDOT@NP with the mechanism inspired by three-terminal organic electrochemical transistors.^14^ An overpotential of ±0.9 V is used, within the potential window to avoid water splitting and adequate to drive ET based on the redox features characterized in Fig. 1 and Fig. 3. PEDOT@NP is at low*-σ* states after cross-linking. Under +0.9 V electrolysis in A­(i), the current increases gradually from tens of nA to about 200 nA over 40 s, followed by sharp switching to high*-σ* states up to 20 μA over 200 s and a gradual increase over time thereafter. The main transition takes a total charge of 1.3 mC. The process is reversed by −0.9 V electrolysis in B­(i). Switching back from high*-σ* states, tens of μA to sub-μA in tens of seconds, consumes about 0.2 mC. Possible explanations for more charges required in the oxidation electrolysis include side reactions of the oxidized bipolarons^48^ and more likely the leakage charges, both electrons and/or (de)­doping counterions diffusing out of the transport-limiting nanotip region. The latter is intuitively conceptualized that it would require less, e.g., a single breaking point, to “disconnect” a circuit or lower the conductance but a continuous framework for boosted conductance across the whole transport trajectory/zone (note that the effective region in the conical nanotip limiting charge transport is not conserved). Notably, the spiking observed in Fig. 5A (enlarged in Fig. S11) correlates with a drastic change in conductivity followed by rapid recovery. This phenomenon occurs exclusively during the early electrolysis stage—characterized by competing doping and dissipation processes—and disappears upon the completion of oxidation once a stable, high*-σ* state is reached. The intermittent conductance fluctuations, alongside the stochastic spiking in Fig. 3B and transient-to-persistent switching behaviors in Fig. 3C, are reminiscent of higher order complex functions emerging from multiple coupled state variables.

**Figure 5:**
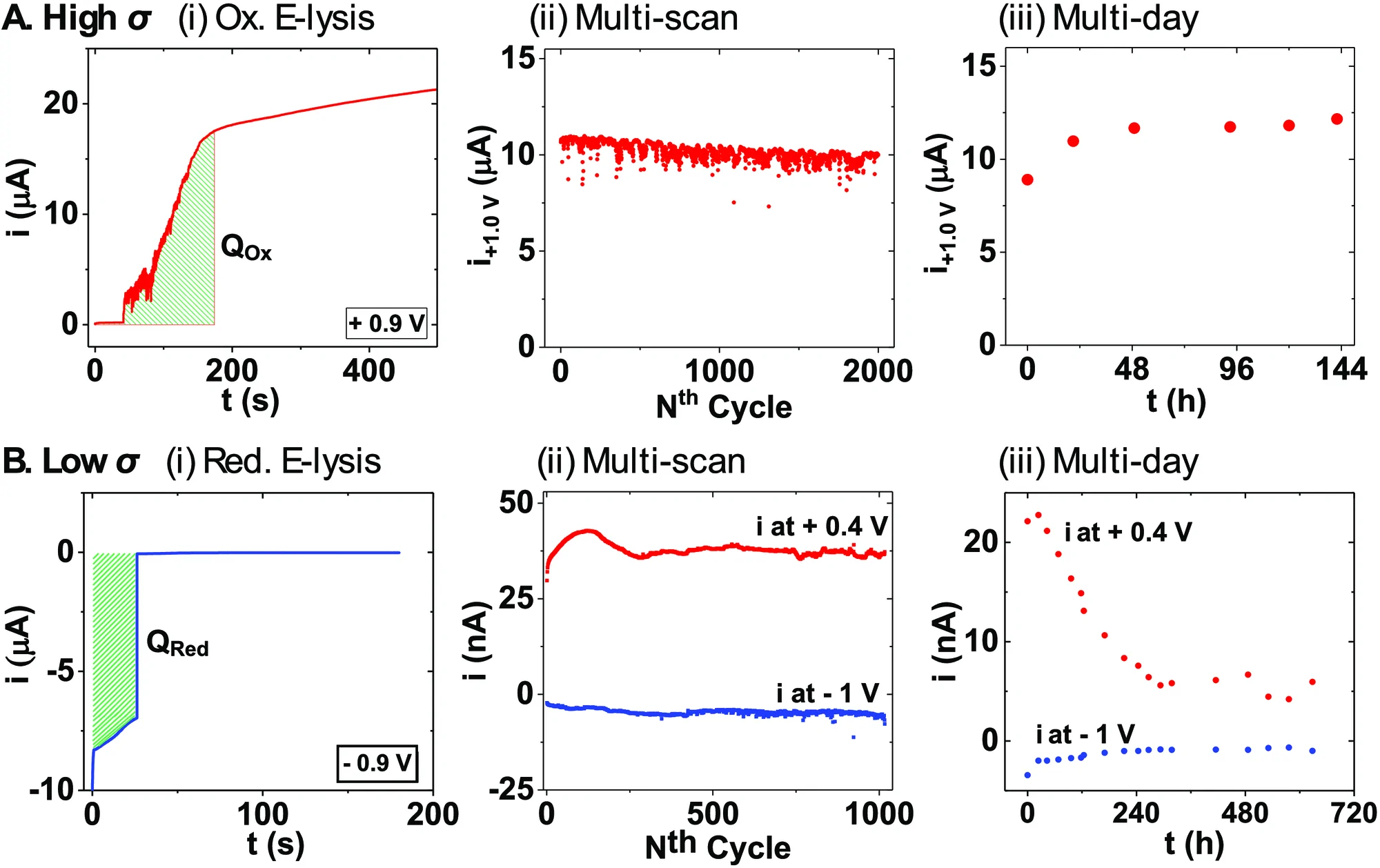
PEDOT@NP locked at (A) high*-σ* and (B) low*-σ* states after electrolysis under constant potentials. (i) current–time traces during oxidation and reduction (Ox./Red. E-lysis) under (A) +0.9 V and (B) −0.9 V using the same PEDOT@NP device. Shaded areas indicate the integrated charges (i*t = *Q*_Ox_ or *Q*_Red_) to approach different high/low*-σ* states. (ii) and (iii) threshold current in the CV scans between (A) 0.0 V to +1.0 V and (B) −1.0 V to +0.4 V over multiple cycles and different hours/days. The scan rate is 0.2 V/s. Data in (ii) are from *I–V* curves in Fig. 3D. The data collection rate is 100 ms/pt for *i*–*t* traces in (AB) (i). Adaptive spiking in A­(i) is enlarged in Fig. S11.

Importantly, the current and charges during electrolysis, driven by the selected potential and duration, should be viewed as counterparts of the gate electrode processes in electrochemical transistors, not the dynamic IT through nanopores. It would therefore be obvious that different *σ* states can be reached in principle by choosing the duration and/or potential during electrolysis for analog or digital applications. The current–time trends under constant potential also corroborate well with the results in Fig. 3C, in which the potential is scanned cyclically. Fundamentally, the charge (current X time), instead of current alone, governs the high–low*-σ* state switching, be it under constant, sweeping or pulsed potential waveform. For reference, much slower switching is observed in Fig. S12 under a pulse train stimulation, or Write.

Multiple PEDOT@NPs are electrolyzed into high- and low*-σ* states separately to evaluate their respective stability and consistency. These devices are tested periodically over hundreds to thousands of repeated cycles (ii) and over days/weeks (iii) following the strategy demonstrated in Fig. 3D. Remarkably, these PEDOT@NPs remain at the high- and low*-σ* states when probed using a narrower potential range, retaining ICR, hysteresis, and NDR features consistently! Despite the initial drift and intermittent noises, which are not unusual for such delicate measurements over a long time span, and until the nanotips and/or electrical contacts are broken after repeated testing, insignificant conductivity changes over days to weeks under ambient conditions unequivocally demonstrate the sustainable and reversible long-term memory (LTM) effects.

### Mechanistic Discussion

Fig. 6 illustrates the correlation of volatile IT features at low*-σ* states (A) with the nonvolatile switching governed by faradaic ET (B). The diode and memristor properties at low*-σ* states (Ai) can be readily explained by the dynamics of concentration polarization at the conical nanopore effective volume region (Aii), sketched qualitatively following previous reports.^19,33,42,53^ Briefly, the high molecular weight of PSS makes cations the main mobile charge carriers. The continued enrichment (or depletion) of charge carriers, or hysteresis in the redistribution of polarized concentration under varying potential, establishes the pinched *I*–*V* loops. The hysteresis in releasing those enriched charges while the system is transiting from HC to LC states induces NDR, while higher scan rates shorten the time for enrichment/depletion and thus weaken the ICR, hysteresis loops, and NDR.

**Figure 6:**
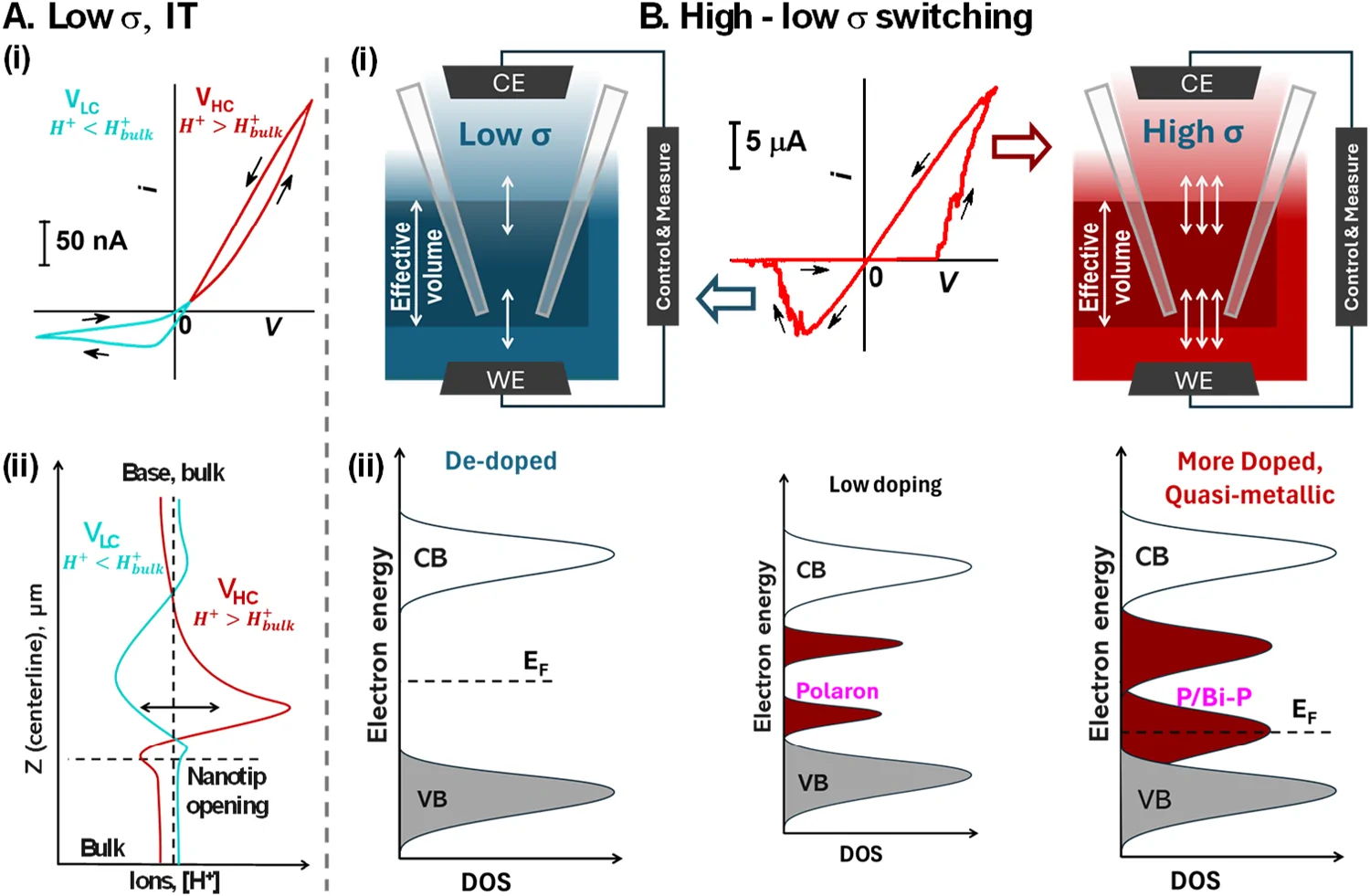
Schematics of mechanism for IT- and ET-regulated conductivity switching in PEDOT@NP. (A) IT in the low*-σ* state (i) rectified pinched *I*–*V* plot and (ii) concentration polarization along nanopore centerline cations as main charger carriers. (B) Switching between oxidation-induced high*-σ* and reduction-induced low*-σ* states. (i) Nanotip at low- and high-conductivity states with the corresponding *I*–*V* plot in the middle and (ii) energy diagram of neutral, polaron, and bipolaron (left to right) PEDOT states. Concentration profiles (A)­(ii) and density of states (DOS, (B)­(ii)) are sketched qualitatively. Note that the Fermi level (E_F_) in the band structure of conducting polymers is sketched near the low-lying frontier states and its location varies with the level of P-doping; voltammetric peaks in conducting polymers are generally broadened with hysteresis in significant deviation from the diffusion-limited solution features.

Most critical to this work, the orders-of-magnitude conductance changes driven by ET (B)­(i) arise from the transition between semiconducting states and a semi-metallic regime as illustrated in the energy diagram (B)­(ii). The corresponding molecular origin is PEDOT redox states and the nanosized phase domains within the PSS framework, at neutral to polaron and ultimately bipolaron depending on the applied potential. Oxidative hole doping introduces polaron/bipolaron bands, which reduces interband transition energy, and the doping levels alter the density of states. In other words, the concentration of PSS^–^ groups is not expected to change during the redox cycle. Rather, the oxidation and/or reduction of PEDOT modifies the doping level and the associated counterion concentration, where ion selectivity would arise from factors such as localized ion pairing and coordination chemistry. Eventually, the band gap may diminish below the thermal energy level, establishing metallic states.^60–62^ Accordingly, the low and high thresholds for state switching depend on the respective conductivity of PEDOT:PSS, more specifically the doping level, which also depends on material synthesis–assembly. Spectroelectrochemistry results in Fig. S13 further demonstrate the transition of PEDOT between neutral and (bi)­polaron states under electrolysis.

## Conclusions

Overall, faradaic ET regulates the IT across PEDOT:PSS-derivatized conical nanopores in which memristor and diode properties result from space charge distribution and nanogeometry confinement. Two sets of state variables, *f*_ET_ and *f*_IT_, describe the ET and IT, respectively; both depend on the extent of PEDOT conjugation, space charge distribution, and polymer-phase domains under nanoconfinements. Two orders of magnitude differences in conductivity states controlled by (de)­doping enable long-term memory up to hours and stability for days, in addition to short-term volatile features. Additionally, the high density of volumetric space charges and electronic conductive PEDOT domains introduced by crosslinked PEDOT:PSS overcomes the flux limitations of traditional surface charge-governed systems. Even when the PEDOT is reduced to its neutral or semiconducting state, these fixed space charges within the PSS framework still regulate counterion migration, providing the diode with ionic rectification, hysteretic, and NDR features. Consequently, this polymer–nanopore platform is actively controlled by space charge-dominated ionic current and doping state-dominant electronic current synergistically. Coupling of ET and IT with the respective intrinsic kinetics and the versatility to separately control their dynamic properties via the sets of state variables, *f*_ET_ and *f*_IT_, provide new avenues to advance neuromorphic computing functionalities, for example, logic operations (diode and controllable switching), in-memory computing (mem-diode, short- and long-term), and in principle spiking/bursting, adaptation, and oscillations (fast and slow dynamics). Much more work is needed, ranging from more quantitative studies on the dynamics of charge carriers to transient spiking and intermittent-to-persistent switching-type functions, which can be rationally anticipated through optimizations on material/device synthesis and measurement control parameters and combining experimental and theoretical studies. Additionally, simulations, in particular, finite element methods for continuum theory and molecular dynamics that can match the experimental time and length scales, represent an important direction to provide insights into the mechanisms underlying the observed properties.

## Supplementary Material



## Notes

Synthesis; experimental details; SEM image and analysis; and additional current–potential data (PDF)

## References

[1] Laughlin S. B., Sejnowski T. J. (2003). Communication in Neuronal Networks. Science.

[2] Laughlin S. B., de Ruyter van Steveninck R. R., Anderson J. C. (1998). The metabolic cost
of neural information. Nat. Neurosci..

[3] Stiefel K. M., Coggan J. S. (2023). The energy challenges
of artificial superintelligence. Front. Artif.
Intell..

[4] Zidan M. A., Strachan J. P., Lu W. D. (2018). The future
of electronics based on
memristive systems. Nat. Electron..

[5] Bou A., Bisquert J. (2021). Impedance Spectroscopy
Dynamics of Biological Neural
Elements: From Memristors to Neurons and Synapses. J. Phys. Chem. B.

[6] Chua L. (1971). Memristor-The
missing circuit element. IEEE Trans. Circ. Theory.

[7] Sangwan V. K., Lee H. S., Bergeron H. (2018). Multi-terminal memtransistors
from polycrystalline monolayer molybdenum disulfide. Nature.

[8] Bae J., Won J., Shim W. (2024). The rise of memtransistors for neuromorphic hardware
and In-memory computing. Nano Energy.

[9] Brown T. D., Zhang A., Nitta F. U. (2024). Axon-like active signal
transmission. Nature.

[10] Di
Ventra M., Pershin Y. V., Chua L. O. (2009). Circuit Elements
With Memory: Memristors, Memcapacitors, and Meminductors. Proc. IEEE.

[11] Lanza M., Sebastian A., Lu W. D. (2022). Memristive technologies
for data storage, computation, encryption, and radio-frequency communication. Science.

[12] Strukov D. B., Snider G. S., Stewart D. R., Williams R. S. (2008). The missing memristor
found. Nature.

[13] Fuller E. J., Gabaly F. E., Léonard F. (2017). Li-Ion Synaptic Transistor
for Low Power Analog Computing. Adv. Mater..

[14] van
de Burgt Y., Lubberman E., Fuller E. J. (2017). A non-volatile
organic electrochemical device as a low-voltage artificial synapse
for neuromorphic computing. Nat. Mater..

[15] Wang S., Chen X., Zhao C. (2023). An organic electrochemical
transistor for multi-modal sensing, memory and processing. Nat. Electron..

[16] Sarwat S. G., Kersting B., Moraitis T., Jonnalagadda V. P., Sebastian A. (2022). Phase-change memtransistive synapses for mixed-plasticity
neural computations. Nat. Nanotechnol..

[17] Waser R., Dittmann R., Staikov G., Szot K. (2009). Redox-Based Resistive
Switching Memories – Nanoionic Mechanisms, Prospects, and Challenges. Adv. Mater..

[18] Kamsma T. M., Boon W. Q., ter Rele T., Spitoni C., van Roij R. (2023). Iontronic
Neuromorphic Signaling with Conical Microfluidic Memristors. Phys. Rev. Lett..

[19] Xiong T., Li C., He X. (2023). Neuromorphic functions with a polyelectrolyte-confined
fluidic memristor. Science.

[20] Zhang P., Xia M., Zhuge F. (2019). Nanochannel-Based Transport in an Interfacial
Memristor Can Emulate the Analog Weight Modulation of Synapses. Nano Lett..

[21] Brown W., Kvetny M., Yang R., Wang G. (2022). Selective
Ion Enrichment
and Charge Storage through Transport Hysteresis in Conical Nanopipettes. J. Phys. Chem. C.

[22] Kamsma T. M., Kim J., Kim K. (2024). Brain-inspired
computing with fluidic iontronic
nanochannels. Proc. Natl. Acad. Sci. U.S.A..

[23] Li C., Xiong T., Yu P., Fei J., Mao L. (2021). Synaptic Iontronic
Devices for Brain-Mimicking Functions: Fundamentals and Applications. ACS Appl. Bio Mater..

[24] Hou Y., Ling Y., Wang Y. (2023). Learning from the Brain:
Bioinspired Nanofluidics. J. Phys. Chem. Lett..

[25] Robin P., Kavokine N., Bocquet L. (2021). Modeling of
emergent memory and voltage
spiking in ionic transport through angstrom-scale slits. Science.

[26] Chen K.-L., Ying Y.-L., Ewing A. G., Long Y.-T. (2026). Nanopipette Electrochemistry. Chem. Rev..

[27] Talin A. A., Meyer J., Li J. (2025). Electrochemical Random-Access
Memory: Progress, Perspectives, and Opportunities. Chem. Rev..

[28] Robin P., Emmerich T., Ismail A. (2023). Long-term
memory and
synapse-like dynamics in two-dimensional nanofluidic channels. Science.

[29] Kumar S., Wang X. X., Strachan J. P., Yang Y. C., Lu W. D. (2022). Dynamical
memristors for higher-complexity neuromorphic computing. Nat. Rev. Mater..

[30] Wang D., Liu J., Kvetny M. (2014). Physical
origin of dynamic ion transport features
through single conical nanopores at different bias frequencies. Chem. Sci..

[31] Schoch R. B., Han J., Renaud P. (2008). Transport phenomena in nanofluidics. Rev. Mod. Phys..

[32] Momotenko D., Girault H. H. (2011). Scan-Rate-Dependent Ion Current Rectification
and Rectification
Inversion in Charged Conical Nanopores. J. Am.
Chem. Soc..

[33] Wang D., Kvetny M., Liu J. (2012). Transmembrane Potential
across Single Conical Nanopores and Resulting Memristive and Memcapacitive
Ion Transport. J. Am. Chem. Soc..

[34] Feng J., Liu J., Wu B., Wang G. (2010). Impedance Characteristics of Amine
Modified Single Glass Nanopores. Anal. Chem..

[35] Sun B., Xiao M., Zhou G. (2020). Non–zero-crossing
current-voltage hysteresis behavior in memristive system. Mater. Today Adv..

[36] Ramirez P., Cervera J., Nasir S. (2024). Electrochemical
impedance
spectroscopy of membranes with nanofluidic conical pores. J. Colloid Interface Sci..

[37] Krems M., Pershin Y. V., Di Ventra M. (2010). Ionic Memcapacitive
Effects in Nanopores. Nano Lett..

[38] Wang D., Brown W., Li Y. (2018). Hysteresis Charges in
the Dynamic Enrichment and Depletion of Ions in Single Conical Nanopores. ChemElectroChem.

[39] Wang D., Brown W., Li Y. (2017). Correlation of Ion Transport
Hysteresis with the Nanogeometry and Surface Factors in Single Conical
Nanopores. Anal. Chem..

[40] Lu H., Xie S., Zhang W. (2025). Negative differential resistance in memristive
systems: historical evolution, mechanisms and neuromorphic applications
of niobium oxide devices. Nanoscale.

[41] Bisquert J. (2023). Iontronic
Nanopore Model for Artificial Neurons: The Requisites of Spiking. J. Phys. Chem. Lett..

[42] Yang R., Balogun Y., Ake S. (2024). Negative
Differential
Resistance in Conical Nanopore Iontronic Memristors. J. Am. Chem. Soc..

[43] Perez-Grau J. J., Ramirez P., Garcia-Morales V. (2021). Fluoride-Induced Negative
Differential Resistance in Nanopores: Experimental and Theoretical
Characterization. ACS Appl. Mater. Interfaces.

[44] Li Z., Myers S. K., Xiao J. (2025). Neuromorphic ionic computing
in droplet interface synapses. Sci. Adv..

[45] Paulo G., Sun K., Di Muccio G. (2023). Hydrophobically gated memristive nanopores
for neuromorphic applications. Nat. Commun..

[46] Kim J. Y., Jung J. H., Lee D. E., Joo J. (2002). Enhancement of electrical
conductivity of poly­(3,4-ethylenedioxythiophene)/poly­(4-styrenesulfonate)
by a change of solvents. Synth. Met..

[47] Takano T., Masunaga H., Fujiwara A., Okuzaki H., Sasaki T. (2012). PEDOT Nanocrystal
in Highly Conductive PEDOT:PSS Polymer Films. Macromolecules.

[48] Rebetez G., Bardagot O., Affolter J., Réhault J., Banerji N. (2022). What Drives the Kinetics and Doping Level in the Electrochemical
Reactions of PEDOT:PSS?. Adv. Funct. Mater..

[49] Wei C., Bard A. J., Feldberg S. W. (1997). Current
Rectification at Quartz Nanopipet
Electrodes. Anal. Chem..

[50] Siwy Z. S., Howorka S. (2010). Engineered voltage-responsive
nanopores. Chem. Soc. Rev..

[51] Hou X., Guo W., Jiang L. (2011). Biomimetic
smart nanopores and nanochannels. Chem. Soc.
Rev..

[53] Baram D., Kvetny M., Ake S., Yang R., Wang G. (2025). Anodized Aluminum
Oxide Membrane Ionic Memristors. J. Am. Chem.
Soc..

[54] Kim Y. D., Choi S., Kim A., Lee W. (2020). Ionic Current Rectification
of Porous Anodic Aluminum Oxide (AAO) with a Barrier Oxide Layer. ACS Nano.

[55] Plett T., Thai M. L., Cai J. (2017). Ion transport in gel
and gel-liquid systems for LiClO(4)-doped PMMA at the meso- and nanoscales. Nanoscale.

[56] Zucker R. S., Regehr W. G. (2002). Short-Term Synaptic Plasticity. Annu. Rev. Physiol..

[57] Bard, A. J. ; Faulkner, L. R. ; White, H. S. Electrochemical Methods: Fundamentals and Applications; Wiley, 2022.

[58] Rivera-Sierra G., Ramirez P., Bisquert J., Bou A. (2025). n. Relaxation Time
of Multipore Nanofluidic Memristors for Neuromorphic Applications. J. Am. Chem. Soc..

[59] Shahrim N. A. A., Ahmad Z., Wong Azman A., Fachmi Buys Y., Sarifuddin N. (2021). Mechanisms for doped PEDOT:PSS electrical conductivity
improvement. Mater. Adv..

[60] De
Keersmaecker M., Lang A. W., Österholm A. M., Reynolds J. R. (2018). All Polymer Solution Processed Electrochromic Devices:
A Future without Indium Tin Oxide?. ACS Appl.
Mater. Interfaces.

[61] Wang Y., Zhu C., Pfattner R. (2017). A highly stretchable, transparent, and
conductive polymer. Sci. Adv..

[62] Qian X., Qiu J., Hu B. (2025). Metal-Like Conductivity in Acid-Treated PEDOT:PSS
Films: Surpassing 15,000 S/Cm. ACS Appl. Mater.
Interfaces.

