# Supplementary material for: Redox-Regulated Nonvolatility Switching in Single-Nanopipette Memory Diodes
Source: J Am Chem Soc. 2026 Sep 19;148(38):41018–27. doi: 10.1021/jacs.6c10813 (PMC13632554; doi:10.1021/jacs.6c10813)
Supplement: Supplementary file 1 [file ja6c10813_si_001.pdf]

Supplementary Materials for

Redox Regulated Non–Volatility Switching in Single Nanopipette Memory-Diodes

Dipak Baram, Ruoyu Yang, Gangli Wang*
Department of Chemistry, Georgia State University Atlanta, Georgia, 30302, United States

*Corresponding author. Email: glwang@gsu.edu

The PDF file includes:

Materials, Devices, Synthesis and Measurements
Figs. S1 to S13 with explanations
References

Materials and Methods

Materials

All materials and reagents were used as received. For nanopipette synthesis: Quartz capillaries; with and without filament, O.D.: 1.0 mm, I.D.: 0.70 mm, 7.5 cm length (Q100-70-7.5, Sutter Instrument Co., CA), Laser puller; P-2000, (Sutter Instrument Co., CA). For solution loading in nanopipette: PTFE tube; ID: 0.008 ± 0.001 inch, wall thickness: 0.004 ± 0.001 inch (Cole Palmer Co., IL), Centrifuge; MC-12 microcentrifuge (Benchmark Scientific Co., NJ). For nanopipette calibration and Poly(2,3-dihydrothieno-1,4-dioxin)-poly(styrenesulfonate) (PEDOT:PSS) crosslinking in nanopipette: Ag wire; dia. 0.25 mm and 0.5 mm, 99.9 % (Thermo Scientific), Pt wire; dia. 0.25 mm and 0.5 mm, 99.9 % (Beantown Chemical), Potassium chloride; 99.0 % (Sigma Aldrich), PEDOT:PSS; *CLEVIOS*™ PH 1000 (Heraeus Epurio LLC, OH), Divinyl Sulphone (DVS); > 96.0 % (TCI America), Dimethyl Sulphoxide (DMSO); 99.9 % (Fisher Scientific), Vortex Genie 2 (Fisher Scientific).

Device fabrication

Nanopipettes were fabricated from quartz capillaries using pulling parameters for ca. 150-nm-radius nanopipettes: line 1: Heat: 700, Filament: 4, Vellocity:55, Delay:180, Pull: 80, line 2: Heat: 700, Filament: 4, Vellocity:60, Delay:150, Pull: 150. The nanopipettes were backloaded using an in-house-made micro-injector constructed with a syringe and PTFE tubing (detailed description in Ref. 21 in the main text) in the sequence of water, KCl solution, water and PEDOT: PSS solution. Centrifugation at 4500 rpm for 12 min was performed after each loading.

PEDOT: PSS crosslinking and in-situ electrochemical monitoring of conductance changes

PEDOT: PSS was crosslinked with some modification following the procedures mentioned in the literature [1,2]. About 500 µL of CLEVIOS PH 1000 PEDOT: PSS solution was mixed with 50 µL water, 50 µL DMSO and 25 µL DVS and vortexed for 5 min followed by 1 h sonication. The mixed solution (~ 12 µL) was backloaded into nanopipettes. The nanopipettes were dipped into an Eppendorf tube containing 400 µL of the rest mixed PEDOT:PSS solution for in-situ conductivity measurements. Two Ag (or Pt) wires, one inside nanopipette and another in the Eppendorf tube outside the nanopipette, were insulated with PTFE tapes with only a short section at the end exposed for electrical connections. The metal wires and quartz capillary were further tied together by wrapping with the PTFE types to ensure robustness in electrical connections during repeated testing. This is referred to as pre-crosslinked sample. The whole assembly was placed in the oven at 60°C for 16-25 h for crosslinking. During the process, the setup was periodically removed from oven at the denoted time points and centrifuged, and electrochemical data was collected at room temperature.

Nanopipette calibration

Prior to polymer derivatization, the nanopipettes were characterized non-invasively by conductivity in 1 M KCl solution following standard procedures in literature [3,4]:

$$R_{\text{cone}} = \frac{1}{\pi \Lambda r \tan\theta} + \frac{1}{4\Lambda r}$$

In this equation, R_{cone} is the Ohmic resistance of the nanopore; Λ is electrical conductivity of the electrolyte; r is the radius of a nanopipette; θ is the half cone angle. An average value of 3.5° is used in the calculation determined by scanning electron microscope (SEM) analysis of multiple nanopipettes prepared using the

same parameters [4]. The term of $\frac{1}{4\Lambda r}$ is access resistance. In 1 M KCl solution (Λ = 10.9 S/m), a linear ohmic *I-V* curve is obtained from which R_{cone} is determined. The linearity also confirms negligible surface effects, warranting volumetric resistance calculation, i.e. the first term of the equation.

SEM and energy dispersive X-ray spectroscopy (EDX) analysis

SEM and EDX data were collected with a Hitachi SU-8230 FE-SEM equipped with cold field emission (CFE) gun (Hitachi High-Technologies Corporation) coupled with Oxford EDS detector. The crosslinked PEDOT-NPs were attached to 35º inclined surface of SEM pin stubs using carbon tape. The pipette tip was further tilted at ~35º to obtain a better tip view image at overall ~70º angle. Side view images were obtained without tilting. A thin Cu wire (dia. ~0.2 mm, length 1 cm) was inserted inside NP and attached to the carbon tape to make better connection between PEDOT and carbon tape on the pin stub to reduce charging effect. High resolution SEM images and EDX spectra were collected at acceleration voltage of 2-5 kV and 10-15 kV respectively. The pressure was maintained at 10^{-4} torr during EDX and SEM data collection.

Electrochemistry and spectroelectrochemistry

All electrochemical measurements were performed using a Gamry Reference 600 (Gamry Co.) potentiostat inside a home-made Faraday cage. Voltametric data were sampled at 1 mV/step, and amperometric monitoring was at 1 ms per point, unless otherwise noted. The UV-visible spectra of crosslinked PEDOT:PSS film were collected using V-770 Double Beam UV-Visible/NIR Spectrophotometer (JASCO Co.) equipped with a monochromator possessing two gratings. PEDOT:PSS film was prepared by drop casting 10 µL PEDOT:PSS suspension onto a plasma treated ITO surface following the same crosslinking procedures described above. The PEDOT:PSS film on ITO was dipped in 0.1 M KCl solution in cuvette and used as working electrode. A Pt foil counter and a quasi-Ag/AgCl wire reference electrode were used. UV-vis spectra with step size 0.5 nm were collected immediately after 4-6 minute-electrolysis at the listed potentials. The change of detector from visible region to NIR region caused artifact offset in absorbance which was corrected manually.

Supplementary Figures

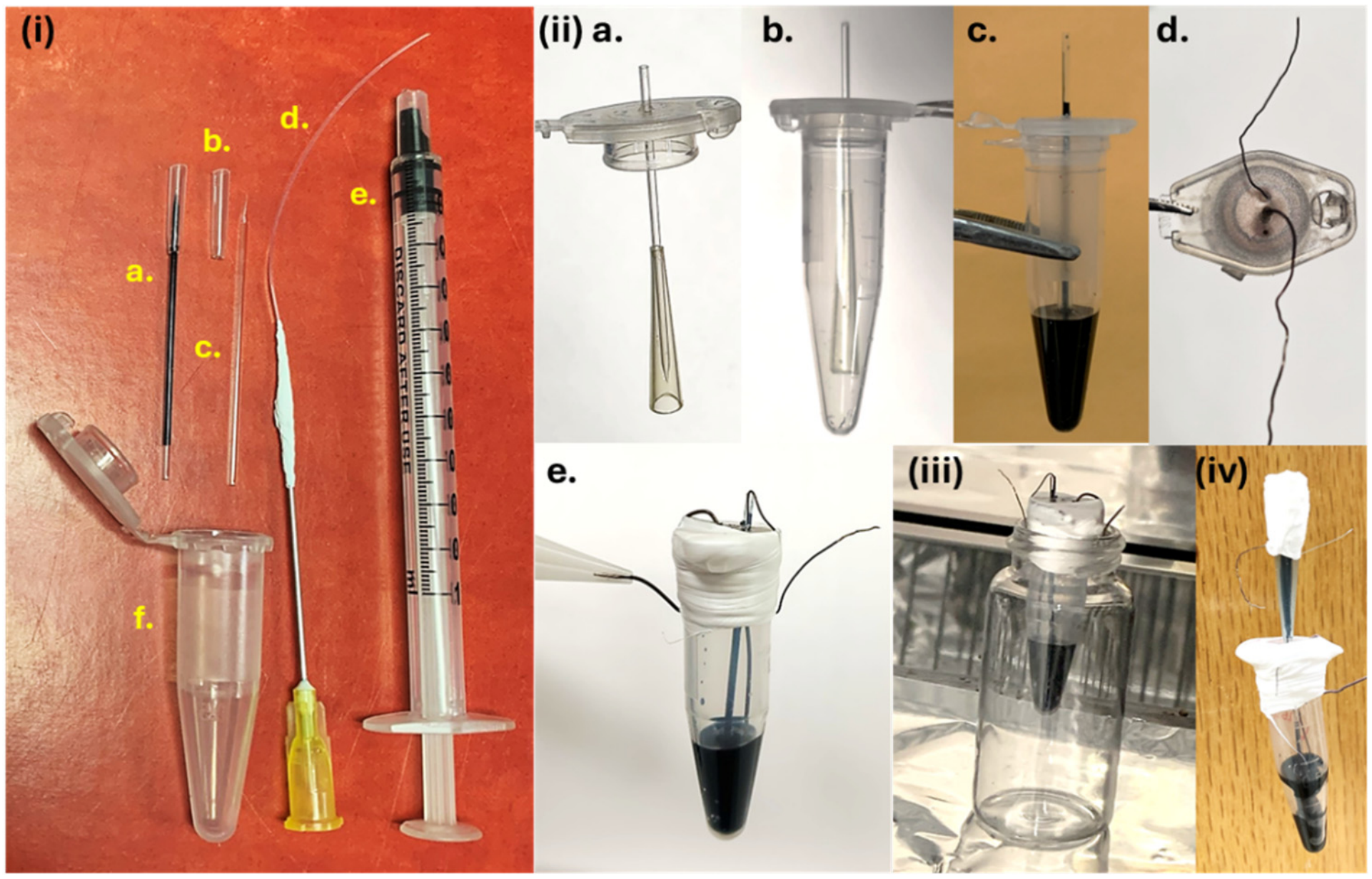

Fig. S1. Procedure and assembly of customized gadgets for handling and measurements of PEDOT@NP. (i) tools for loading the viscous PEDOT solution into the NPs. (a) PEDOT loaded NP with inserted tip guard; (b) tip guard made with 200 µL size pipet tip, cut to nudge-fit with the outer diameter of the quartz capillary; (c) empty NP; (d) syringe needle with PTFE tube secured with PTFE film to load solution into NP; and (e) 1 mL syringe. (ii) Stepwise procedure of NP loading. (a) NP with tip guard back inserted into Eppendorf tube cap; (b) guard alignment inside the Eppendorf tube; (c) PEDOT loaded NP inserted into the same PEDOT solution; (d) top view of electrode wire insertion; (e) fully assembled PEDOT@NP after fixture of exposed metal wire with PTFE tape. (iii) glass vial with PEDOT@NP assembly heating in autoclave for crosslinking process. (iv) 4E system set up for electrochemical measurements. Another pipet tip is connected to the top of NP fully loaded with PEDOT solution for addition space to make electrode insertations.

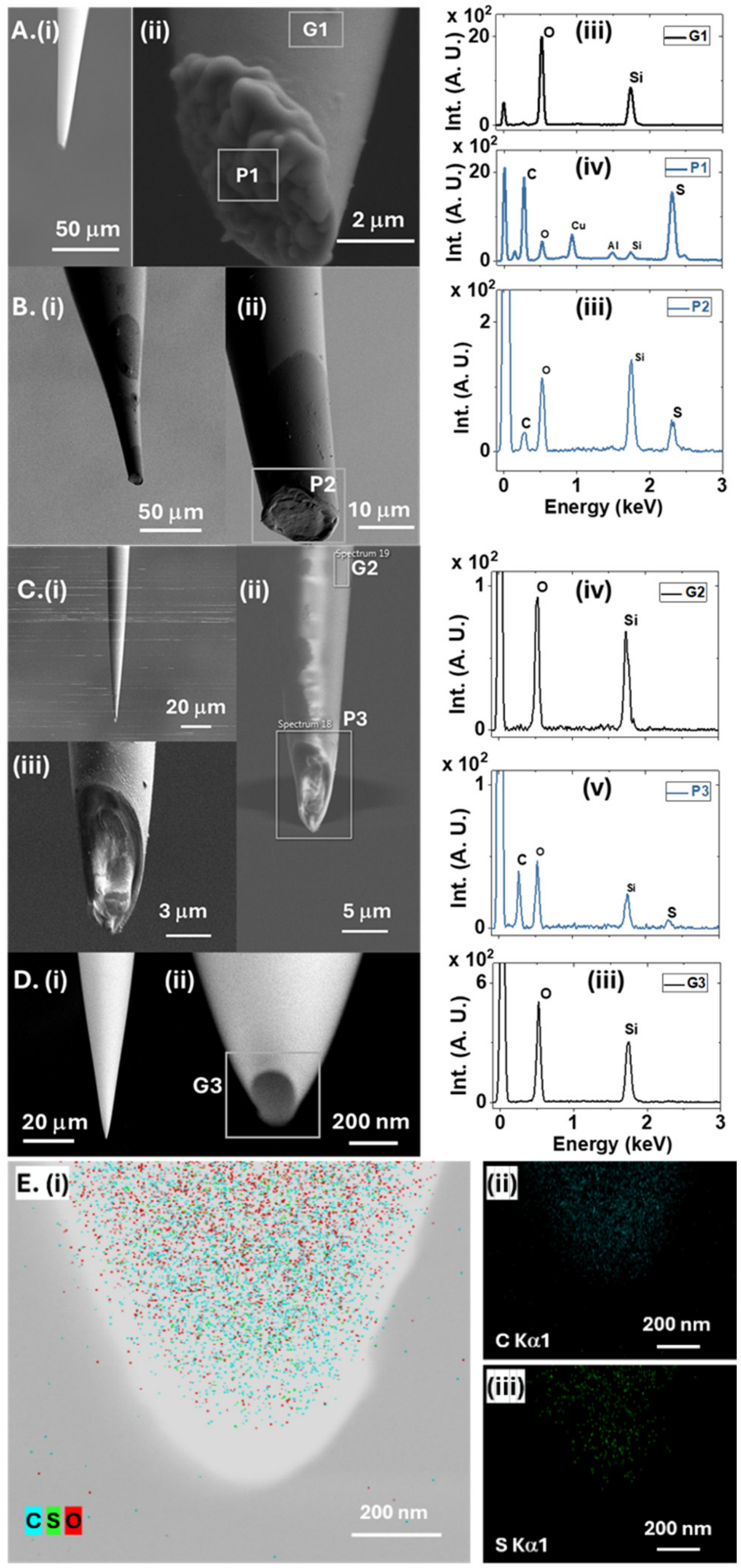

Fig. S2. **SEM and EDX analysis of additional intact and broken nano-micro-tips.** (**A, B,** and **C**) correspond to three different PEDOT@NPs displaying continuous filling of PEDOT inside the stem of the capillary up to the pipette tips (i) side view and (ii) tip view. Boxes in the images indicate the area for EDX signal integration. Strong S (and C) signals in reference to the SiO_2 from quartz capillary appeared in EDX (**A**) (iv), (**B**) (iii), (**C**) (v) of P1, P2 and P3 region, but not in G1 and G2 which are away from the tip shown in (**A** iii) and (**C**) (iv). (**D**) Empty nanopipette without PEDOT (i) side view and (ii) tip view. S and C signals were not detected in the empty pipet tip region G3, (**D**) (iii). (**E**) EDX mapping of PEDOT@NP nanotip (from Fig1 (Bi)) showing co-distribution of (i) carbon, sulphur and oxygen, (ii) carbon only and (iii) sulphur only.

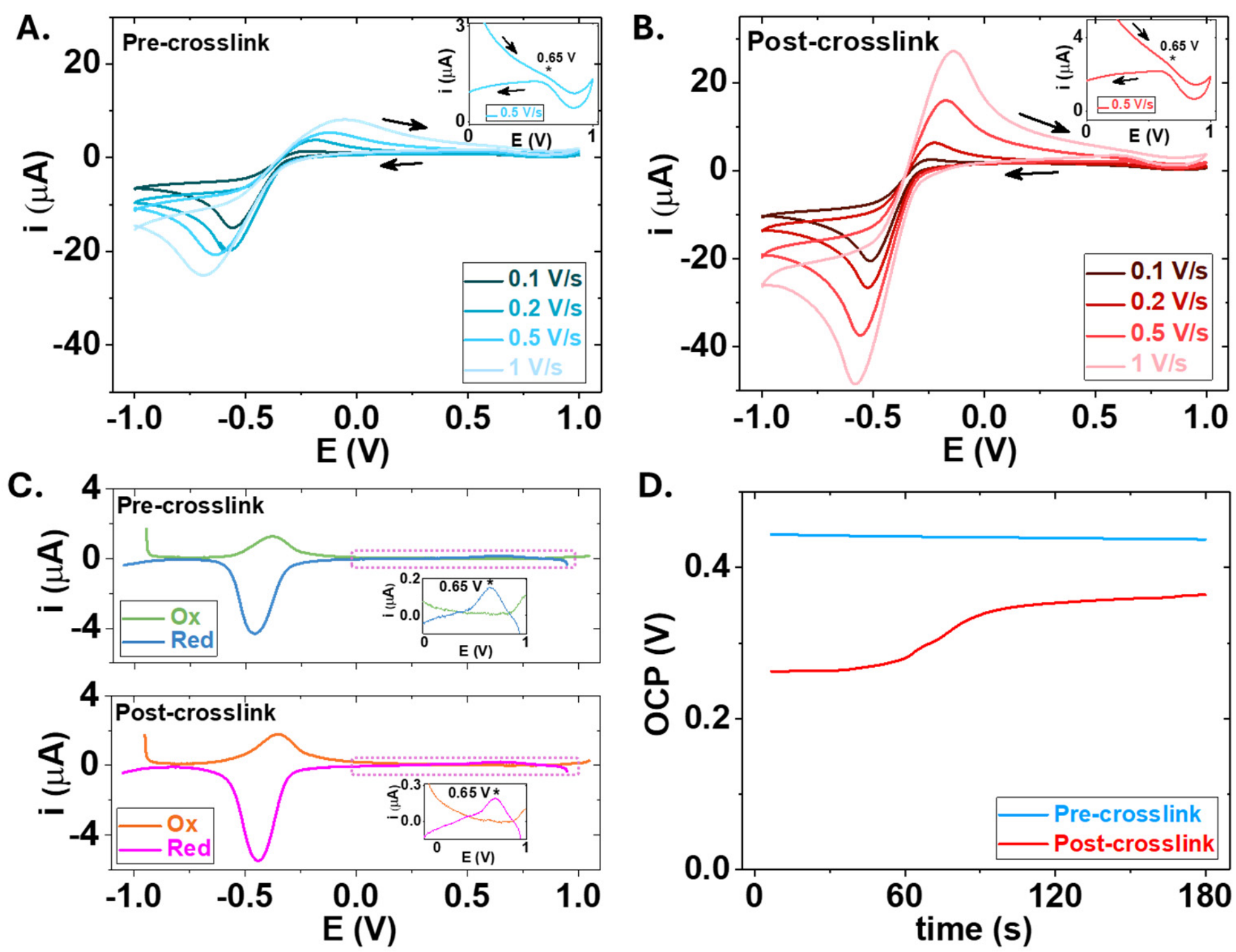

Fig. S3. Voltametric features of PEDOT@NP. Panels (**A**) and (**B**) provide CVs of pre- and post-crosslinked PEDOT:PSS at additional scan rates. Panels (**C**) and (**D**) are differential pulse voltammograms and open circuit potential analysis. Insets are enlarged view around the second anodic features at + 0.65 V. Data were collected with three-electrode setup using Pt disk (dia. 0.5 mm) as working electrode, a Ag/AgCl (saturated KCl solution) reference, and a Pt foil counter electrode. Increased peak separation between the cathodic peak around - 0.5 V and the reversal anodic peak at higher scan rate is qualitatively indicative of a less reversible redox process. However, electron transfer in polymer liquid and films involves multiple intertwined factors and should not be treated using diffusion limited theorem [5]. This topic is beyond the scope of this work and will be separately pursued in a follow-up work.

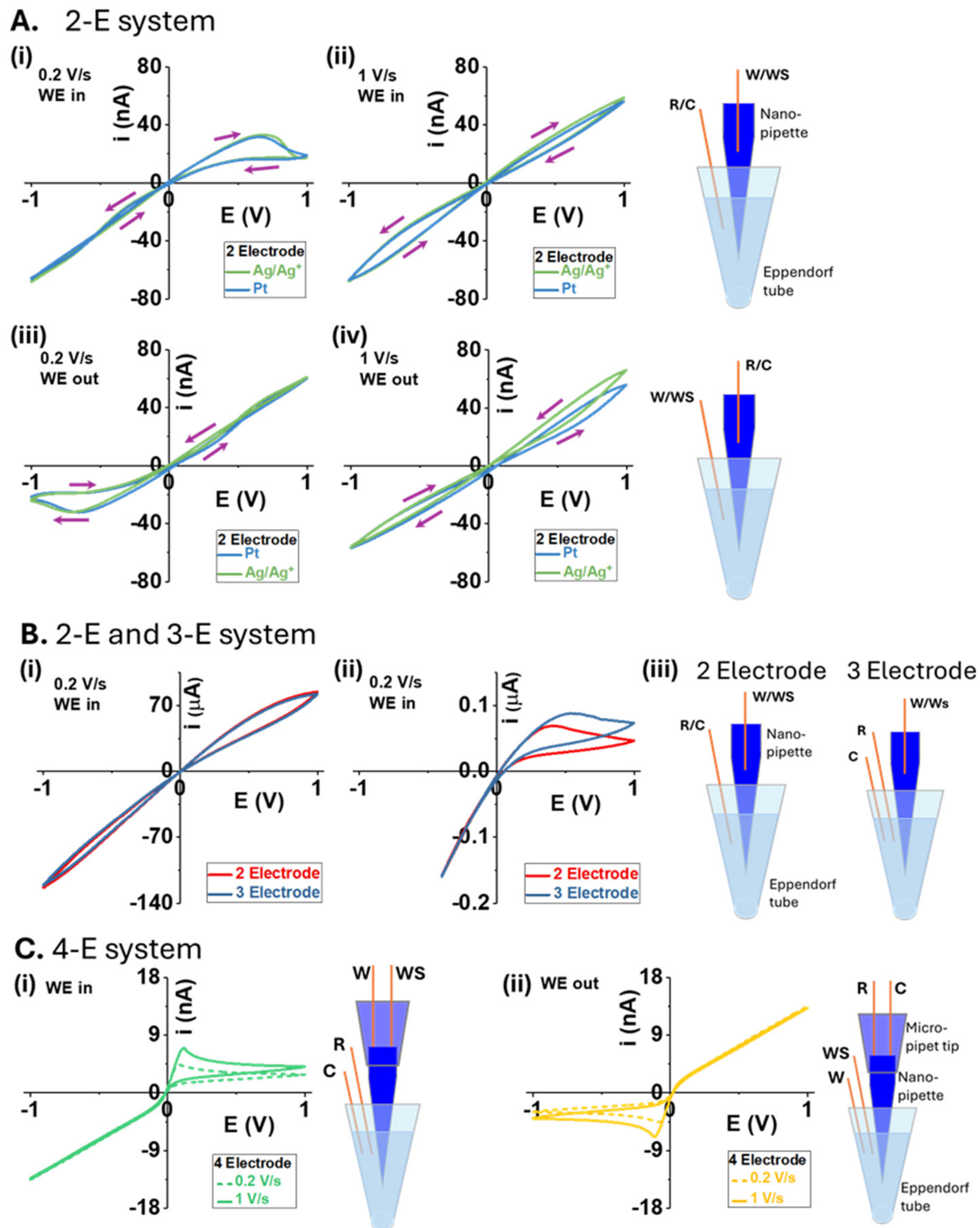

Fig. S4. CVs with different electrode configurations for the validation of *I-V* features. The electrode arrangements are sketched on the right for each type of control experiments. All *I-V* data were acquired from pre-crosslinked PEDOT@NP (so that electrodes can be replaced readily) except data in (**B**). (**A**) Comparisons of *I-V* data collected with two Pt wires vs two Ag/AgCl wires as working-working sense (W/WS) and counter-reference (R/C) electrodes in 2-Electrode setup. (i) and (ii) are WE inside the nanopipette at 0.2 V/s and 1 V/s, (iii) and (iv) are WE outside at 0.2 V/s and 1 V/s respectively, data obtained from same NP. (**B**) Comparison of *I-V* plots obtained with 2-Electrode versus 3-Electrode setup in (i) high σ conductivity state and (ii) low σ conductivity state of crosslinked PEDOT. The schematic of electrode setup is in (iii). (**C**) 4-Electrode setups with Pt wire as WE and CE for current to flow through; and Ag/AgCl wires as RE and WS for potential control and recording in pre-crosslinked PEDOT@NP. WE and WS are (i) outside and (ii) inside of nanopipette. To perform the 4E experiments, an additional micropipette tip was connected to the quartz capillary through the back end of nanopipette, so that two electrode wires can be inserted separately.

From (**A**), the *I-V* features, specifically ICR and hysteresis are similar irrespective of different electrode material (Pt and Ag). Those subtle differences are insignificant relative to the current drift and other experimental uncertainties. In other words, differences in polarizable or nonpolarizable working electrode are insignificant, indicating the recorded current is limited by IT through the nanotip instead of ET at the WE.

From (**B**), compared to the 2-E system where both WE and RE/CE are Pt wire, a separate Ag/AgCl wire as RE is added in 3-E system. Separating RE and CE should allow more current to flow (through CE) while the potential is more accurately controlled by the Ag quasi reference electrode. The current is almost identical at high σ state which again suggests negligible influence by electrode materials within the time scale of measurements. The higher current in LC states (positive potential range) with separate CE and RE (versus shorted CE-RE) outside at low σ state is somewhat unexpected and requires further deliberation. Regardless, the qualitative trend of ICR and hysteresis remain unchanged which adequately support the discussions in the main text.

From (**C**), additional and separate polarizable and nonpolarizable electrodes in both sided made no distinctive differences in the ICR and hysteresis in *I-V* plot, compared to other electrode setups. The results validate the data from 2-E configuration which greatly simplifies the measurements. Lastly, change in ICR polarity (A) (i) vs (iii), (ii) vs (iv) or C. (i) vs (ii) is readily understood with respect to the change in WE position inside vs outside.

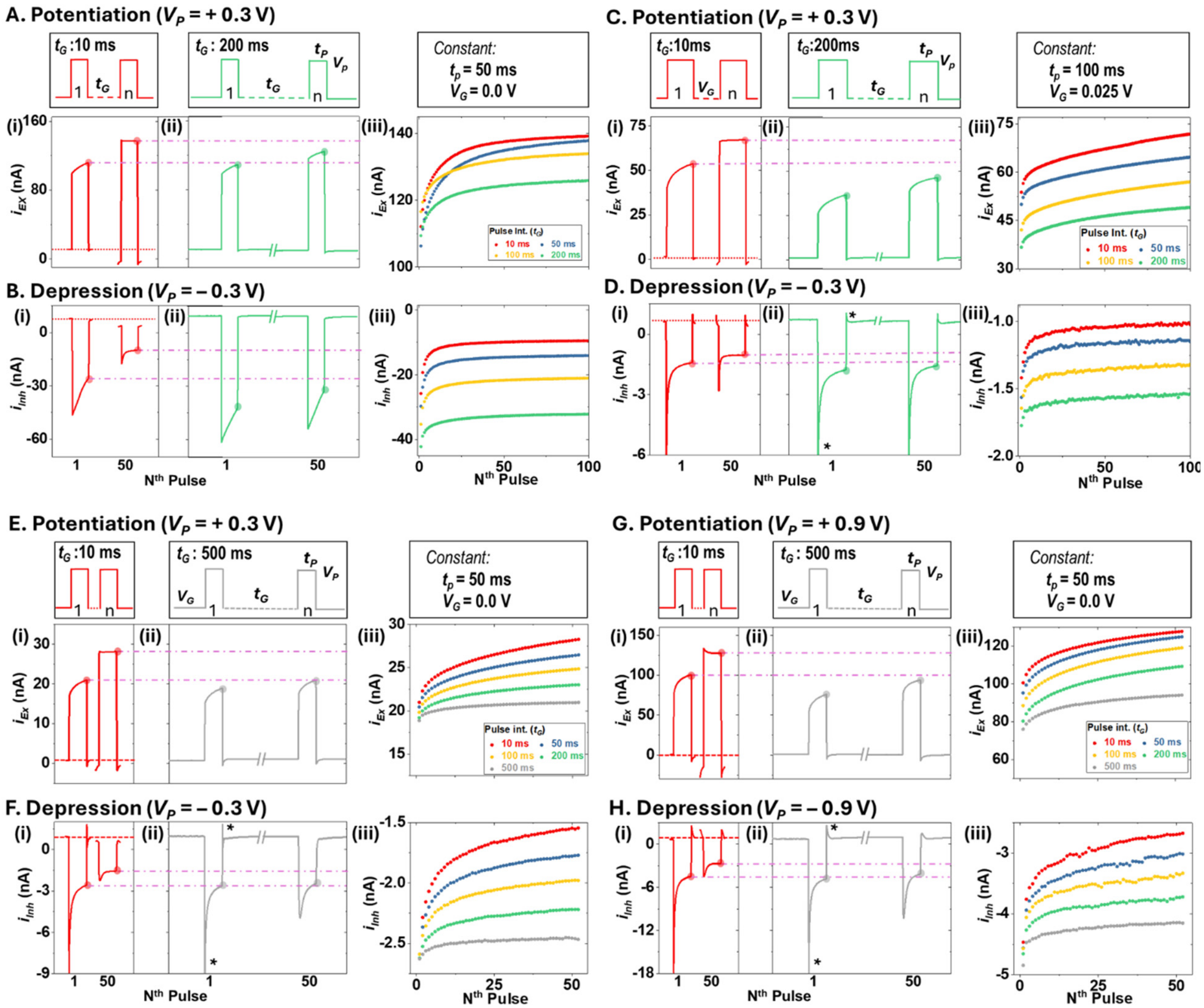

Fig. S5. Additional results with varied pulse parameters and from different PEDOT@NPs. The pulse waveform is sketched on the top for excitatory (**ACEG**, pulse potential (V_P) + 0.3 V and + 0.9 V) and inhibitory (**BDFH**, V_P - 0.3 V and – 0.9 V) conductance features. The pulse duration (t_P) is 0.05 s except **CD** (0.1 s). The potential is 0.0 V during pulse intervals/gaps (t_G) except **CD** (0.025 V which is the crosspoint potential in CV). At 1 ms per point, current during pulse #1 and #50 with intervals of 10 ms and 200 ms in (**A**) - (**D**) and 10 ms and 500 ms in (**E**) – (**H**) are enlarged respectively in (i) & (ii) to illustrate current changes in individual pulses. The trend over repeated pulses is shown in (iii) by plotting the last points in each pulse against pulse number. These exemplary data were obtained with three NPs: (**AB**) from one, (**CD**) from another, and (**EFGH**) and Fig 2. from third NPs. For data in **ABCD**, no preconditioning was applied. A preconditioning of 10s at 0.0 V was used for data in **EFGH**.

Note: high σ states cannot be accessed under the short pulses, e.g. while collecting data in GH. This is because PEDOT@NPs are in low σ states after crosslinking and short pulse electrolysis is inadequate to oxidize the PEDOT to form the highly doped conductive domains.

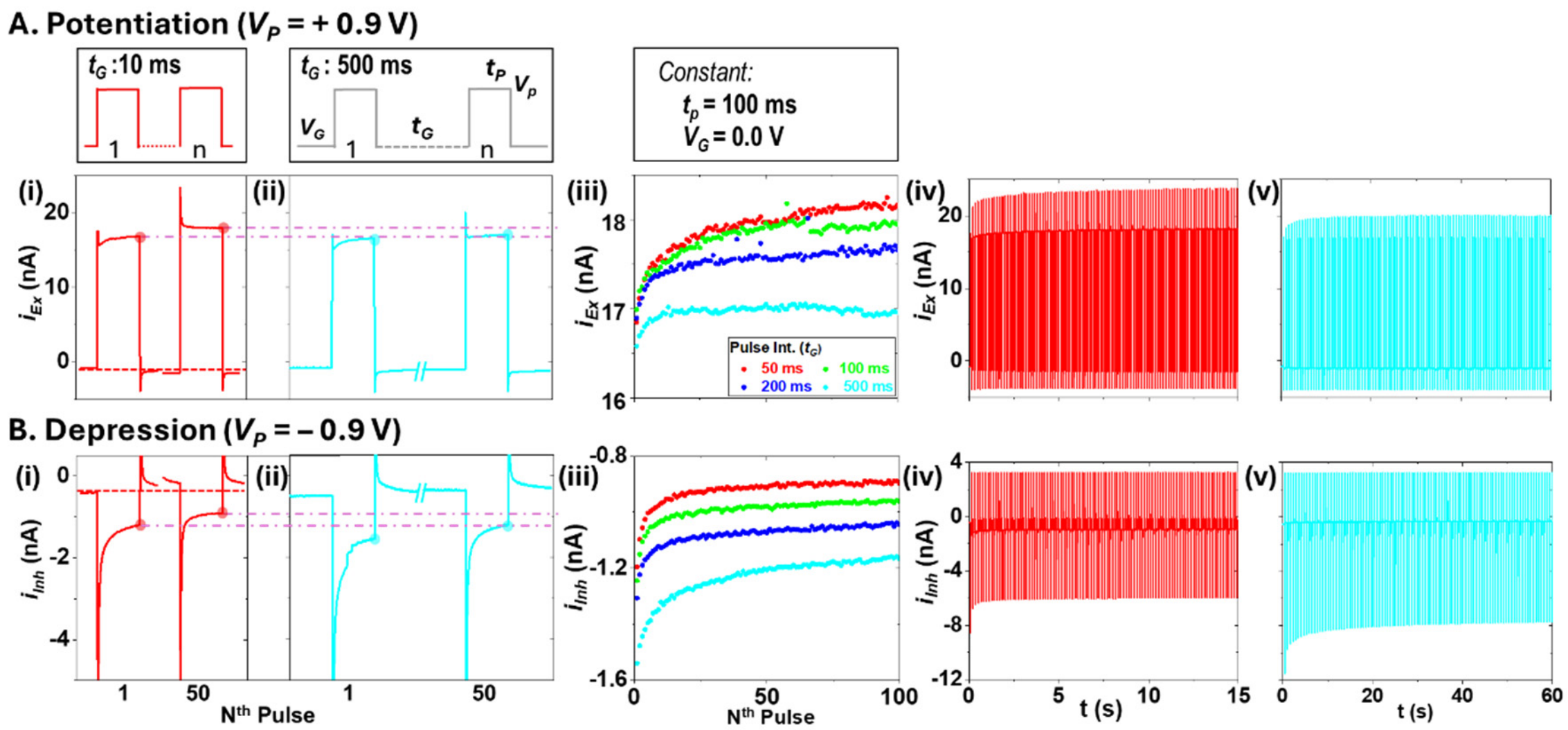

Fig. S6. Excitatory (A) and inhibitory (B) conductance features from non-crosslinked PEDOT@NP. The pulse waveform is sketched on top, with pulse height of +/- 0.9 V and duration (t_P) is 0.1 s. The potential was 0.0 V during pulse intervals/gaps (t_G). The current was recorded at 1 ms per point, pulse #1 and #50 with intervals of 50 ms and 500 ms are enlarged in (i) & (ii) respectively to illustrate current changes in individual pulses. The trend over repeated pulses is shown in (iii) by plotting the last points in each pulse against pulse number. Full current-time traces for (i) and (ii) were provided in (iv) and (v) for reference. No preconditioning was applied.

Note: without crosslinking, high σ states cannot be accessed. The short-term plasticity (STP) behaviors follow the same trend as those of normal nanofluidic memristor behaviors.

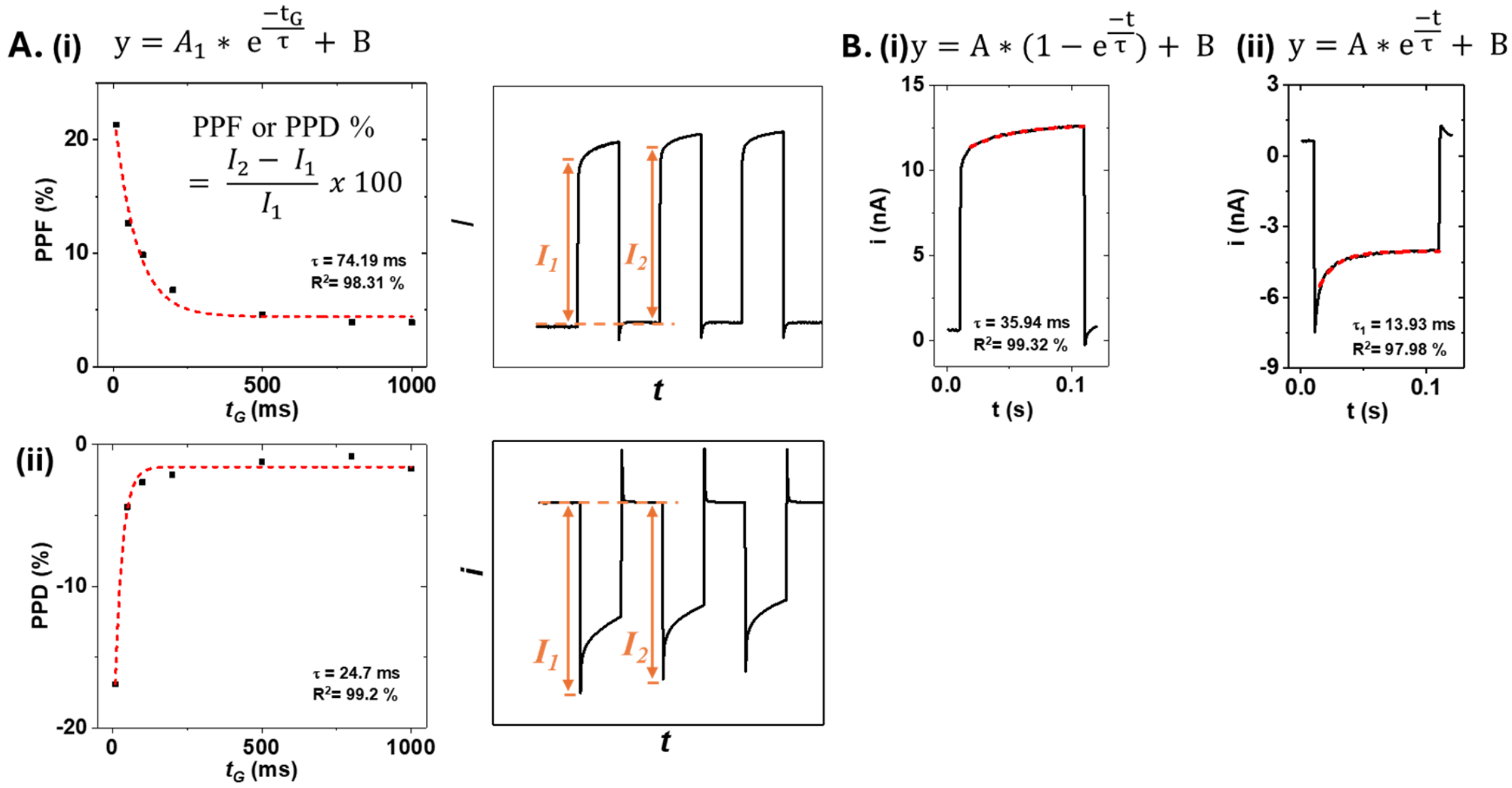

Fig. S7. Fittings of the PPF/PPD and individual current curves in one pulse. (**A**) (i) Paired pulse facilitation (PPF) and (ii) depression (PPD) analysis of data from Fig. 2 AB. The PPF/PPD were calculated from the first and second pulses, shown on the right. (**B**) Fitting of representative *i-t* curves within one pulse during (i) facilitation and (ii) depression. Data from the first pulse of Fig. 2 AB (i). Fitted result is indicated with red dashed lines based on the equations listed on the top.

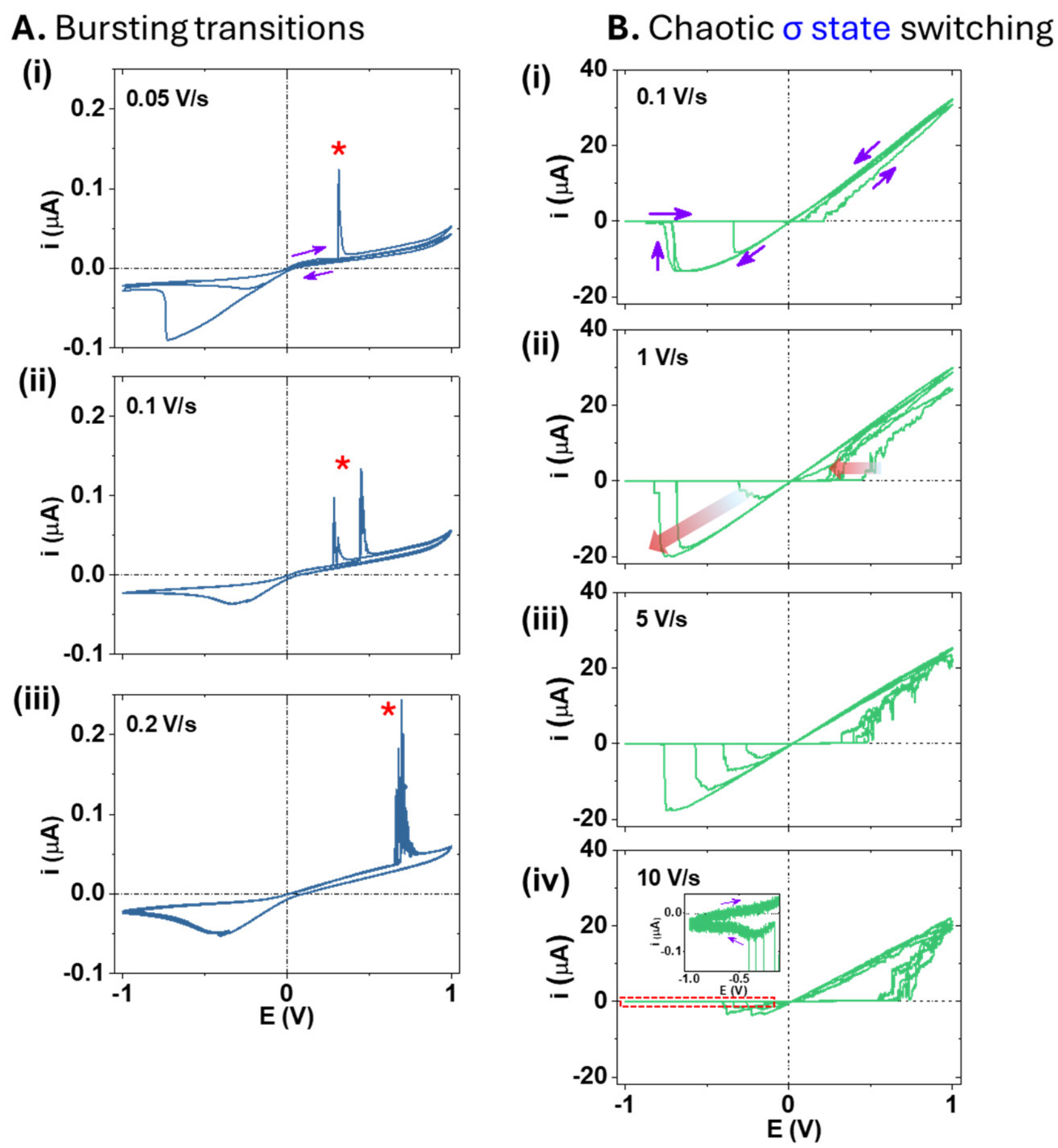

Fig. S8. Another PEDOT@NP displaying transient transitions and high-low σ state switching. (**A**) Transient current spikes (indicated by red asterisks) at low σ state toward the end of crosslinking (16-18 hrs). Three *I-V* cycles are shown at 0.1 V/s and 0.2 V/s, 2 cycles in 0.05 V/s. Arrows in panels (i) show the directions of potential scan and current loop including NDR in negative potential range. (**B**) Discrete conductivity switching after complete crosslinking. Note that current at low σ states is not zero and reminiscent of those in (**A**), see magnified *I-V* of red dotted box in (iv). Block arrows in (ii) indicate trends with increasing scan cycles (4 cycles shown).

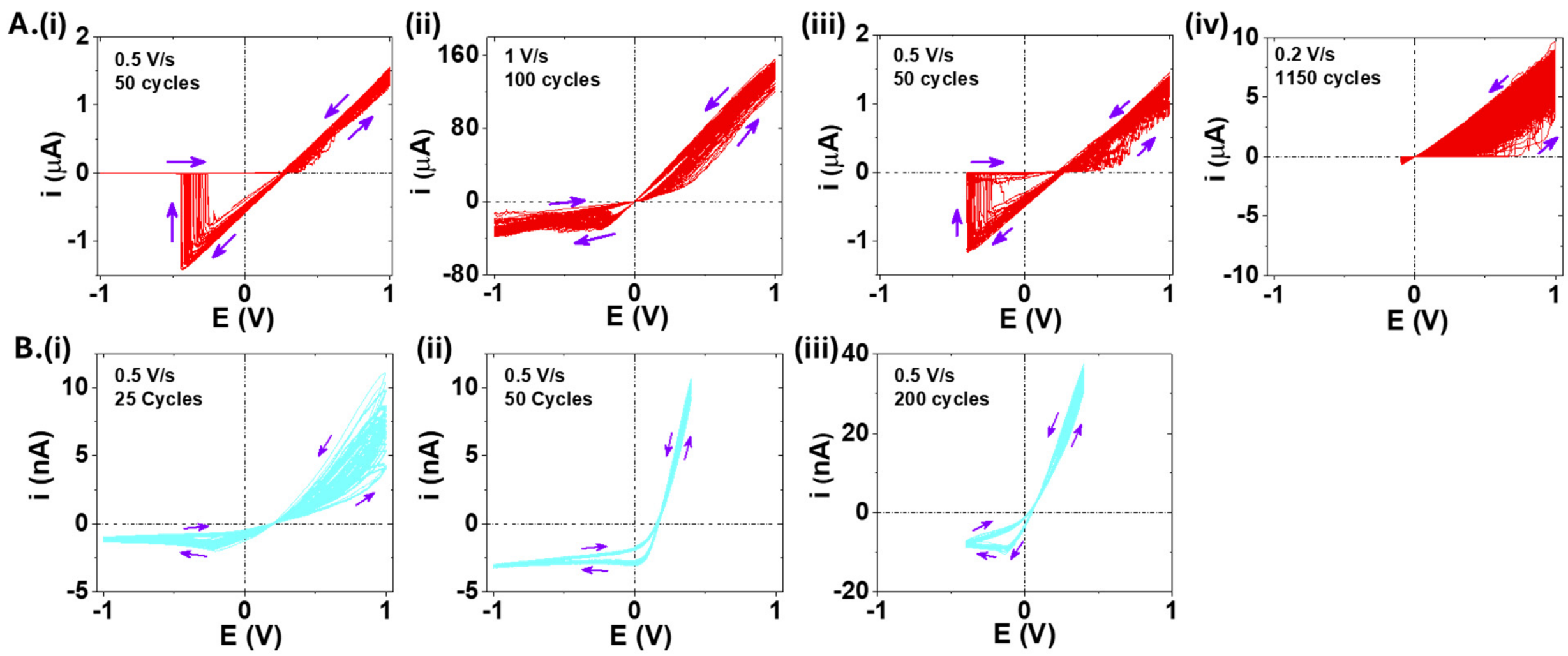

Fig. S9. Switching behaviors over tens-hundreds cycles. (**A**) Stochastic switching events at high σ state at different scan rates and potential ranges. (i) and (ii) - 1.0 V to + 1.0 V at different scan rates; and retaining high σ state by narrower negative potential range of (iii) - 0.4 V to + 1.0 V and (iv) - 0.1 V to + 1.0 V). (**B**) No-to-limited switching from low to high σ state by varying the potential window. (i) - 1.0 V to + 1.0 V, (ii) – 1.0 V to + 1.0 V and (iii) - 0.4 V to + 0.4 V).

Note that current at high conductivity state is in µA range and low conductivity state at nA range. Panel B (i) is an expection where no switching was observed. This is explained by inadequate doping during oxidation and that low σ state is generally more stable and persistent compared to high σ state.

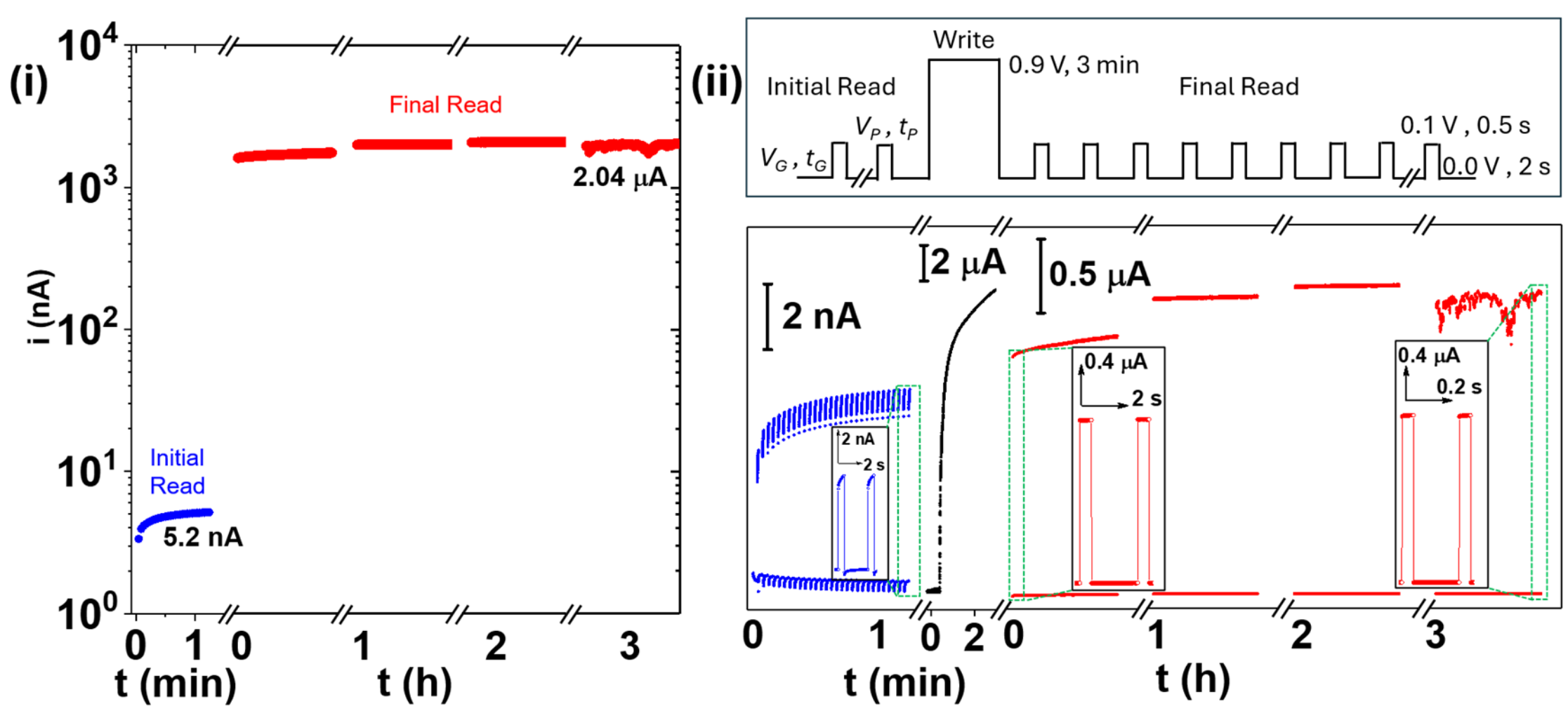

Fig S10: Illustration of long-term memory (LTM) in PEDOT@NP. i) the last point of Read current in each pulse vs time, ii) programmed Read-Write-Read pulse waveform (top panel) and the correponding current (in dots) over time traces (bottom panel). Three insets are enlarged views of the last two pulses from Initial Read, and the first and last two pulses from the Final Read. Note the scales for current and time axis are different for the three steps; and Initial and Final Read are separated by breaks from current during Write at much higher potential bias. The final read pulse was collected for five minutes and waited for an hour without applying potential before collecting another one. The three one-hour intervals are indicated by the

breaks 1, 2 and 3 (//) in (i) and (ii) for the comparison with the consecutive Final Read presented in main text. System was brought to low conductivity state by electrolysis at -0.7 V for 61 s prior to collecting initial read pulses. Step size is 10 ms per point.

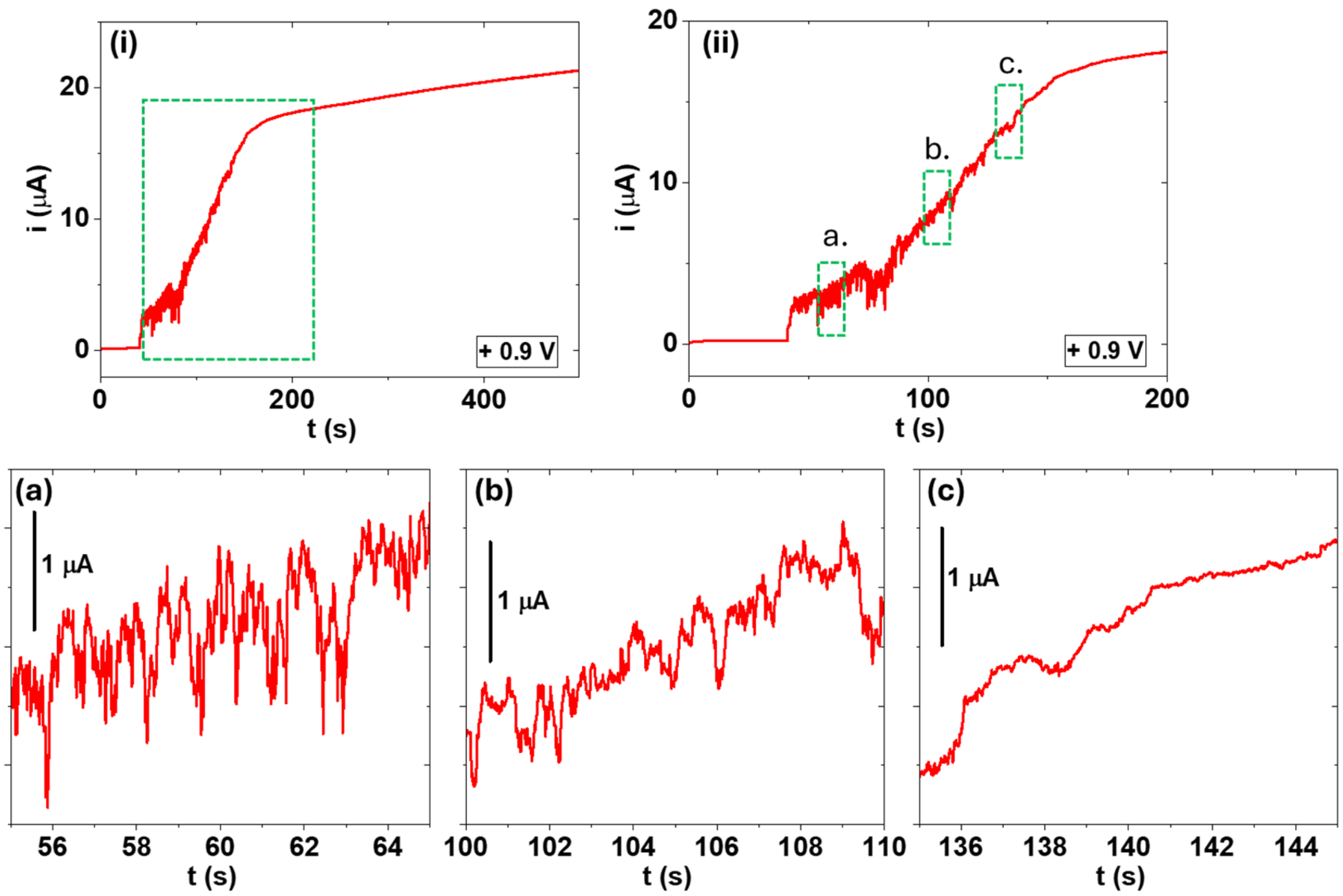

Fig. S11. Adaptive spiking (oscillations) in PEDOT@NP. (i) High conductivity state switching during electrolysis at 0.9 V; (ii) enlarged view of early current increase (green rectangle area in (i)); and further enlarged in (a) to (c). The frequency and magnitude of those current spikes are higher initially and decrease over time, signature of adaptation qualitatively. Data from Fig. 5. (A).

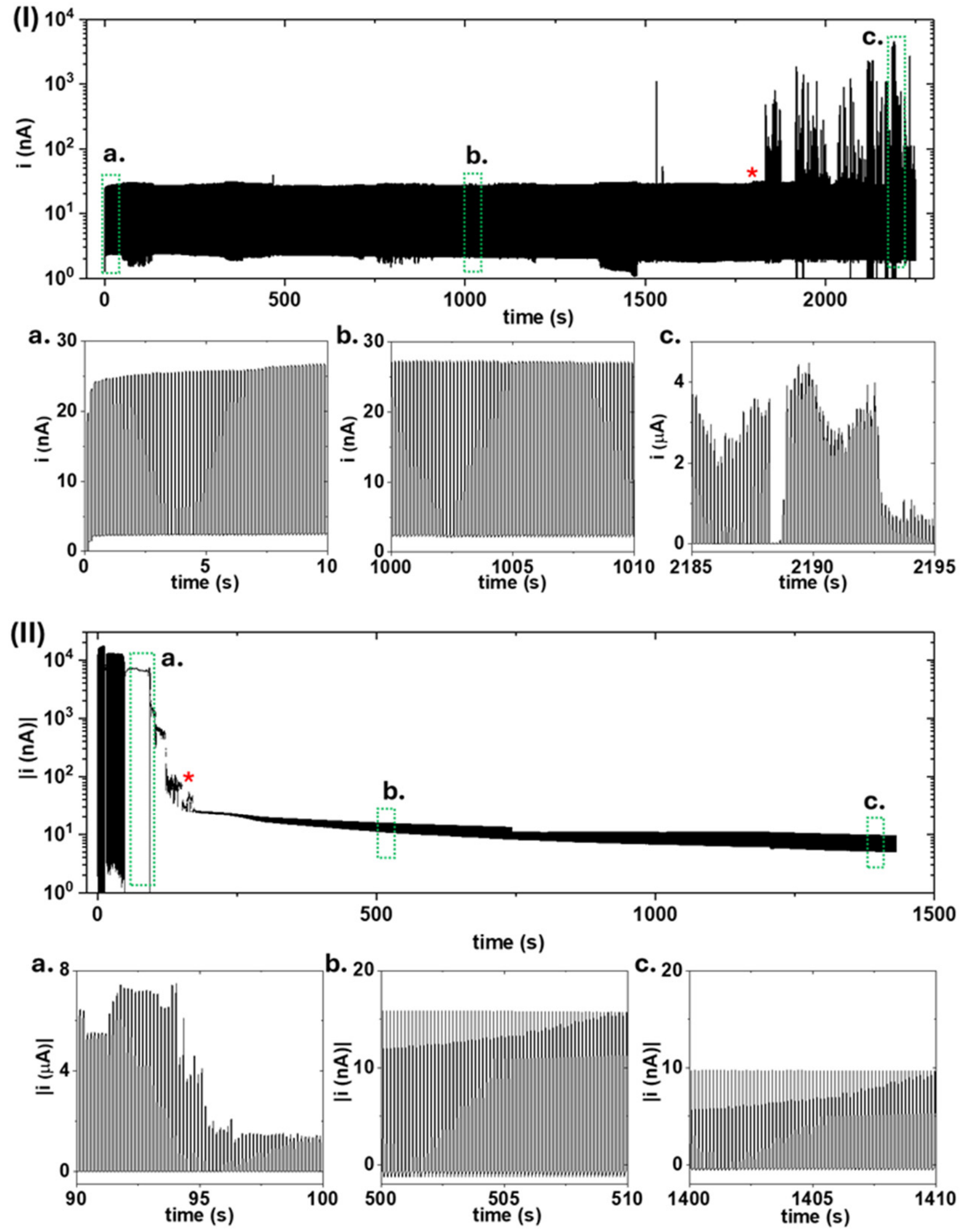

Fig. S12. High – low conductivity state switching under pulse train stimulation. The potential was stepped between V_G = 0.0 V for 100 ms (t_G) and **(I)** V_P = + 0.9 V **(II)** V_P = - 0.9 V for 50 ms (t_P). Current is in log scale due to the large changes, over about 40 mins in (i) and 24 mins in (ii). Panels (a) to (c) are enlarged view. Red asterisks indicate the initial switching to the low/high σ states. Current sample rate was 10 ms per point. High σ state current in µA and low σ state current in nA units. (I) and (II) were obtained from different NPs.

Doping and de-doping via oxidation and reduction are interrupted during the gap period in pulse train stimulation. The corresponding state switching is thus delayed compared to continuous electrolysis under constant potential. The switching is partial and intermittent, again the adaptive bursting due to the dynamic transitions between the original and the more doped and conductive states. As explained in the main text, these observations can be intuitively conceptualized that it would require less, e.g. a single breaking point, to 'disconnect' a circuit or lower the conductance, e.g. the doped PEDOT domains, but a lot more for continuous circuit for boosted conductance across the whole transport trajectory/zone.

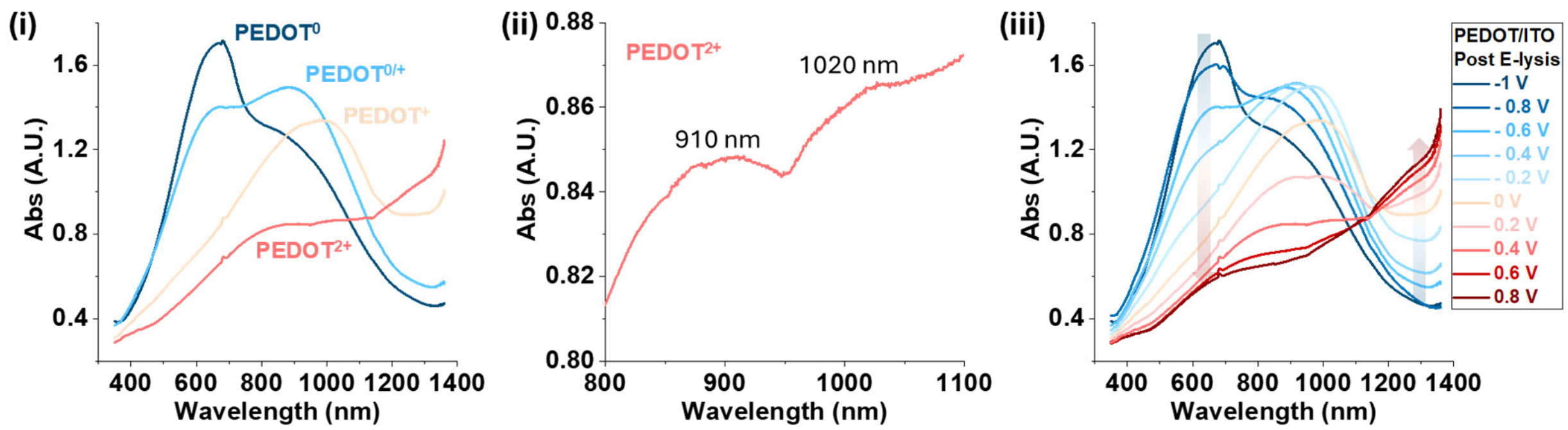

Fig. S13. Spectroelectrochemistry analysis of PEDOT doping effects. (i) UV-Vis spectra of neutral ($PEDOT^0$), polaron $PEDOT^+$ and bipolaron ($PEDOT^{2+}$) (blue to red). A mixed polaron and neutral ($PEDOT^{0/+}$) spectrum is also included that is reminiscent of the as synthesized PEDOT:PSS. (ii) Magnified bipoaron spectrum with two resolved peaks and (iii) evolution of different oxidation states under electrolysis at different potentials.

References:

1 Mantione, D. *et al.* Low-Temperature Cross-Linking of PEDOT:PSS Films Using Divinylsulfone. *ACS Appl Mater Interfaces* **9**, 18254-18262 (2017). https://doi.org/10.1021/acsami.7b02296

2 del Agua, I. *et al.* DVS-Crosslinked PEDOT:PSS Free-Standing and Textile Electrodes toward Wearable Health Monitoring. *Advanced Materials Technologies* **3** (2018). https://doi.org/10.1002/admt.201700322

3 Yu, R.-J., Ying, Y.-L., Gao, R. & Long, Y.-T. Confined Nanopipette Sensing: From Single Molecules, Single Nanoparticles, to Single Cells. *Angewandte Chemie International Edition* **58**, 3706-3714 (2019). https://doi.org/https://doi.org/10.1002/anie.201803229

4 Brown, W., Kvetny, M., Yang, R. & Wang, G. Selective Ion Enrichment and Charge Storage through Transport Hysteresis in Conical Nanopipettes. *The Journal of Physical Chemistry C* **126**, 10872-10879 (2022). https://doi.org/10.1021/acs.jpcc.2c02051

5 Daum, P. & Murray, R. W. Charge-transfer diffusion rates and activity relationships during oxidation and reduction of plasma-polymerized vinylferrocene films. *The Journal of Physical Chemistry* **85**, 389-396 (1981). https://doi.org/10.1021/j150604a017
